# Supplementary material for: Smart event-triggered MINFLUX microscopy to catch and follow rare events
Source: Nat Commun. 2026 May 21;17:4558. doi: 10.1038/s41467-026-73176-z (PMC13195108; doi:10.1038/s41467-026-73176-z)
Supplement: Supplementary file 1 — Supplementary Information [file 41467_2026_73176_MOESM1_ESM.pdf]

Supplementary information

Smart event-triggered MINFLUX microscopy to catch and follow rare events

†Jonatan Alvelid[1,2,3], Agnes Koerfer[1,2], †Christian Eggeling[1,2,4]

[1] *Institute for Applied Optics and Biophysics, Friedrich Schiller University Jena, Jena, Germany*

[2] *Leibniz Institute of Photonic Technology, Jena, Germany*

[3] *Department of Applied Physics and SciLifeLab, KTH Royal Institute of Technology, Stockholm, Sweden*

[4] *Jena Center for Soft Matter, Jena, Germany*

† Corresponding authors: Jonatan Alvelid (jonatan.alvelid@scilifelab.se), Christian Eggeling (christian.eggeling@uni-jena.de)

List of content

1. **Supplementary Notes**
2. **Supplementary Figures**
3. **Supplementary Tables**
4. **Supplementary References**

List of Supplementary Notes

1. EtMINLUX widget
2. EtMINFLUX control widget in comparison with etSTED
3. Scanning-dependent confocal-MINFLUX coordinate shifts
4. Napari widget for etMINFLUX data visualization
5. Real-time analysis pipelines
6. Analysis pipeline parameter optimization and performance evaluation
7. Data throughput comparison between etMINFLUX and manual acquisition control
8. MINFLUX track data filtering steps in caveolae analysis
9. Diffusion analysis on 2D and 3D MINFLUX tracking data
10. Geometrical analysis of endosomal vesicles
11. Z-artefact filtering of 3D MINFLUX tracking data
12. Simulated Brownian motion and MINFLUX tracking
13. EtMINFLUX data handling

List of Supplementary Figures

1. etMINFLUX widget GUI
2. Further experimental modalities enabled by event-triggered MINFLUX
3. Scanning-dependent confocal coordinate shifts
4. Stability of Caveolin1 spots in a confocal timelapse
5. Schematics of analysis pipelines
6. Analysis pipeline performance
7. Exemplary dataset metadata value distributions
8. Caveolin site data analysis sketch
9. etMINFLUX caveolae and random sites with SM-STAR RED – further examples
10. etMINFLUX caveolae and random sites with DPPE-STAR RED – further examples
11. Temporal data throughput for Caveolin1 experiments
12. MINFLUX localization precision
13. Diffusion analysis on simulated data
14. Diffusion coefficient and transient diffusion coefficient analysis results from Caveolin1 accumulation sites
15. Packing coefficient analysis of SM and DPPE diffusion
16. Full MINFLUX datasets of previous plots with subsampled MINFLUX plots
17. etMINFLUX endocytosis sites – further examples
18. Pipeline performance evaluation
19. Live imaging of detected Dynamin1- and comparison to Dynamin1+ sites
20. etMINFLUX endocytosis site with long endosome neck
21. Diffusion coefficient and transient diffusion coefficient analysis results from Gag accumulation sites

22. etMINFLUX virus budding sites – further examples
23. Live imaging of detected Gag- and comparison to Gag+ sites
24. Manual Gag+ event site recording

List of Supplementary Tables

1. etMINFLUX analysis pipeline parameter value ranges
2. Confocal and MINFLUX acquisition parameters in the various experiments

Supplementary Note 1. EtMINFLUX control widget

The etMINFLUX control widget is a software module written in Python using a view-controller design pattern. The control widget was developed to be self-standing and interacts with the Abberior MINFLUX microscope control software Imspector. The widget is entirely open source and available on GitHub, and is available in two versions for either Imspector version m2410 (main m2410 version) or version m2205 (m2205). The latest m2410 version at the moment of submission of this manuscript is also available as an executable (v1.2.0).

The control widget of the two versions interacts with Imspector using various combinations of three means: the specpy package[1], which is a Python-based API for Imspector released by Abberior Instruments; the mouse package[2], which allows user-like emulation of mouse movements; and pynput[3], which allows user-like emulation of keyboard interaction. The occasional reason for the need of the latter two is missing commands in the specpy API for MINFLUX-specific controls, due to a lack of a package version after MINFLUX microscope release. While effort by abberior in the m2410 version of Imspector now allows only minimal and non-critical usage of mouse and pynput, ultimate integration of still missing methods in the API would allow mouse and pynput to be completely removed and allow for a more user-friendly widget implementation. It is important to note that the required version of specpy, v1.2.3, is only available with the latest versions of Imspector on abberior MINFLUX microscopes (see GitHub repository for more information on specpy installation). The publicly available version v1.2.1 does not contain the required functionality for control of MINFLUX acquisitions.

In the m2410 version, a .json file is required to set specific default values and hardware availability for the specific MINFLUX microscope to be used by the user. In this dictionary, available lasers and detectors can be setup; default values for laser powers, ROI sizes, and etMINFLUX parameters can be set; timing delay values for the code can be set; a data saving folder can be set; available MINFLUX sequence names can be limited to a certain subset of interest for the user; and a simulated environment for etMINFLUX can be requested. The .json file, together with the default analysis pipelines, are initiated in the .../Documents/etMINFLUX/ folder upon first launching the software. Modifications should be made to these files to affect the widget and analysis pipelines.

The control widget is built up of individual subwidgets, allowing the user to control what and how information is displayed during etMINFLUX experiments: the main widget, a calibration widget, an event detection overview widget, and an event view widget. The main widget has controls for the real-time analysis pipeline, the experiment mode in use, how the real-time analysis is run, the MINFLUX acquisition, the binary mask recording, and various data saving

modalities. The real-time analysis pipeline control starts with choosing which pipeline to load, after which all the defined parameters for the pipeline shows up in the GUI with editable fields. This allows the user to tweak the parameter values to the sample and experiment at hand. Before running an experiment, the user can choose to record a binary mask using the positive and negative thresholds and smoothing options. The binary mask will be sent to each analysis pipeline call and can be applied in order to limit the area that is considered for the analysis.

After setting the analysis pipeline parameters, the recording mode of the etMINFLUX method can be set using two separate controls. Firstly, the user can change between experiment, visualization test, and validation test modes. The former runs the experiments as normal, while the latter two are two different modes that can be used while not triggering MINFLUX acquisitions. Here, the confocal imaging is continued as normal and the analysis pipeline is run, but as soon as an event is detected, the pipeline either continues to run the confocal as normal while in visualization mode, or the experimental event data is saved, including a set of confocal images that are recorded after the event is detected, which allows the user to further validate the event detection in post-acquisition analysis. In the visualization mode, the detected events are marked on top of the latest confocal image in the event detection overview widget, allowing the user to easily see what is being detected as events and allows a fast feedback for optimizing and tweaking the analysis pipeline parameters. In this widget, a list of all the events with their pixel positions and region of interest (ROI) IDs is further shown. The list can be interacted with by the user and coming ROIs to be recorded can be deleted, for example if the event detection was false.

For normal experiment runs, the second set of recording mode controls allows the user to control the ROI following modes. Here, the user can activate ROI following, i.e. interleaved MINFLUX and confocal measurements of an event site after event detection, and chose the type of ROI following mode: single-site, multi-site, single-site with redetection, and multi-site with multi-detection. Two further parameters can be set: the MINFLUX acquisition time per ROI per cycle, as well as a redetect distance threshold that will be used in the redetection mode that will keep running the analysis pipeline in the interleaved confocal images after event detection and will update the event site position for each MINFLUX cycle. The latter recording mode is particularly useful for an event site that can move over time. When it comes to the multi-site with multi-detection mode, a time window or threshold for the number of event sites before MINFLUX initiation can be set. Detected event sites are added to the queue of ROIs, which already at this stage can be interacted with, until the set goal is met. When it is met, or the user manually starts the MINFLUX acquisitions, interleaved MINFLUX at the individual event sites and confocal recordings are cycled, so as to follow the development at all sites simultaneously.

The analysis control part of the widget further allows control over how the analysis pipeline will be run. Confocal data is immediately upon acquisition pushed to the control widget, a process controlled using line update signals and thus data is accessible on a line-by-line basis. The base case is to run analysis after each recorded confocal frame, however it can optionally be set to run after a set number of lines, the analysis period, if faster event detection is required. Furthermore, a pause between confocal frames can be set, if slower event appearance is expected; a number of initial frames to ignore can be set; and an option to show the event

detection overview widget while running the experiment mode can be set. In ROI following modes the latter defaulted to be on and allows direct interaction with the list of event ROIs where they are continuously sorted according to the next ROI in line to be recorded, in the case of multiple ROI following, and individual ROIs can be deleted by the user to allow full focus on the events of interest.

Lastly, before running an experiment, the MINFLUX acquisition parameter can be set from the etMINFLUX main widget. As MINFLUX acquisition parameters otherwise requires to enter the MINFLUX mode of Imspector, these cannot be set preemptively by the user directly in Imspector. Instead, the MINFLUX sequence, the MINFLUX excitation laser and excitation power, potential activation power, as well as ROI size and acquisition time can all be set in the main etMINFLUX widget. The corresponding values in Imspector will automatically be set by the widget before a MINFLUX acquisition takes place, which also allows the user to tweak these between cycles in the ROI following mode. Boolean controls if to record ROIs in random positions of the binary mask (i.e. random instead of event sites); if to trigger subsequent MINFLUX acquisition of all detected events; and if to use a pre-set ROI size and MINFLUX acquisition time or not are also provided – if not the ROI size needs to be determined by the analysis pipeline, and the MINFLUX acquisition time will be indefinite.

When initiating an etMINFLUX experiment, the experiment can be run in endless mode, which will repeat the process after finishing a MINFLUX recording, allowing the widget to control the automatic experiments indefinitely. When an event recording has finished, the MINFLUX, confocal, and metadata will be automatically saved. An option is provided if the user does not want to automatically save the .msr file with the MINFLUX data, and furthermore an option to automatically delete MINFLUX datasets after an event recording can also be found. When an event is detected, the etMINFLUX will automatically draw a ROI in the right place and initiate the MINFLUX recording.

In the m2205 version, the calibration subwidget hosts options about the screen configuration, which needs to be calibrated in order for the automatic mouse movements to function properly. Here the user needs to detect the top left and bottom right corners of the confocal recording window in Imspector (which needs to be unobstructed during etMINFLUX recordings), after which the widget will automatically read the confocal image size in pixels and micrometers from Imspector, which finally allows a conversion between image position and screen position to be able to draw the MINFLUX ROIs at the right place. There is also a way to calibrate the screen positions of the repeat measurement button and the top MINFLUX datasets in the dataset viewer widget in Imspector, which will be used to click the button and select the top MINFLUX dataset for deletion (if that option is selected) respectively. These three types of functionality are not available in the *specpy* Python API for Imspector m2205 and thus needs to be handled by simulated moues movements and clicks. In the calibration subwidget there are further parameters for various timing options that are needed for proper functioning due to the simulated mouse movement interactions necessary – if drawing the ROI too fast or if clicking the necessary buttons too fast after each other the control is unreliable. For optimal temporal performance, these timings need tweaking between individual microscope control computers as they depend on the computer specifications.

In the m2410 version, using the latest updates to v1.2.3 of specpy in which further functionality of MINFLUX-specific control was added by abberior, most of the calibration mentioned above is not necessary. Instead, etMINFLUX experiments can be run without emulated mouse or keyboard movement or presses, allowing full headless running mode. In order to automatically delete MINFLUX datasets upon finishing of an acquisition, a calibrated GUI position of the MINFLUX dataset list, in the calibration settings subwidget, and mouse and keyboard emulation is still required.

The last subwidget is the event view, that is shown when a MINFLUX acquisition is initiated at an event site. In this subwidget, the user can view a zoomed-in view of the event site over the whole timelapse stack of confocal images recorded. Additionally, a graph is shown plotting the confocal intensity summed in a small area around the event site over time. A dropdown menu allows the user to switch between all detected event sites, and the stacks and graphs are continuously updated during a ROI following experiment, allowing the user to inspect and decide if to keep or delete ROIs before further cycles are recorded.

The simulated etMINFLUX environment available to the user in version m2410 does not require specpy to run, and can be used in order to gain familiarity to the GUI and test and optimize analysis pipelines on pre-recorded and loaded confocal datasets. It is loaded if the user set the system_simulation parameter to True in the setup .json file, or further loads automatically if specpy does not successfully loads or if no connection to Imspector is achieved.

Supplementary Note 2. EtMINFLUX control widget in comparison with etSTED

The etMINFLUX control widget shares many similarities with and is based on the generic event-triggered microscope control widget previously developed as part of event-triggered STED imaging[4]. However, many important key differences exist that required the development of a new control widget.

First and foremost, the event-triggered acquisition concept has here been developed a step further, now allowing various acquisition modes such as multiple ROI detection and ROI following of various forms to be controlled directly from the control widget. In the previously released generalized version, etSTED-widget-base (https://github.com/jonatanalvelid/etSTED-widget-base), in an event-triggered experiment only a single triggering of a high-resolution method was allowed, after a detected event. In etMINFLUX, we implemented different recording modes, such as triggering of multiple high-resolution ROIs from a single monitoring frame, waiting to detect multiple events in a time window, and following a single or multiple ROIs in a timelapse acquisition of both methods, all requiring new functionality to be built in. To control these recording modes, the addition of multiple control parameters was necessary. Additionally, to aid the user during these more complex experiments, additional visualization of the low-resolution timelapse data has also been added, allowing the user to take decisions of continuation or cancellation during the experiment, which is of importance to further increase the throughput of longer ROI follow experiments.

Secondly, as we want to avoid to setup MINFLUX acquisition parameters beforehand directly in Imspector, control for those had to be added in the control widget. Having that control

available only in Imspector would mean a requirement to control both control software, while in the current implementation, no interaction with Imspector is required after loading the measurement template with a confocal recording window. Furthermore, manually setting MINFLUX acquisition parameters in Imspector requires the selection of a MINFLUX ROI and thus entering the MINFLUX recording mode, a process adding additional complexity to the experiments and a larger room for user error. Using the previous etSTED widget, setup of STED acquisition parameters had to be done beforehand directly in the acquisition widget or software.

Thirdly, as etSTED was using camera-based widefield imaging and etMINFLUX uses point-scanning confocal imaging as the monitoring method, the implementation of the monitoring required certain code changes. Reading when an analysis period had finished and triggering the real-time analysis required more functional steps and connection of intra-code signals. Furthermore, the point-scanning allowed the addition of a sub-frame analysis period with which the user now can decide to perform analysis after the acquisition of every N lines, useful if for example looking for extra rapid events and a large and/or slower confocal acquisition is used.

Lastly, as etMINFLUX is implemented to control an external commercial control software, certain signals and control between the two is required, and a whole-new Python module specpy is required for this control. While these changes could have been implemented as an Imspector backend interacting with the old widget, the other additions and adaptations anyway made the overhaul necessary and thus the direct implementation of such software control was made in the Controller-part of the code.

All in all, these developments of the concept, added functionality and thus adapted GUI, and requirements of the control widget implementation made an adaptation of the earlier released etSTED control widget etSTED-widget-base limiting and cumbersome. Instead, borrowing certain code snippets and the main framework but with a large overhaul of the functionality and GUI allowed an easier and better implementation of the here-described event-triggered method. Moreover, since this control widget is developed to control a commercial control software, the aim is that the exact version presented here can be beneficial for many users in the community without modifications to the code. The released executable further strives to meet this aim.

Supplementary Note 3. Scanning-dependent confocal-MINFLUX coordinate shifts

Despite being scanned by the same scanning apparatus and sharing a global coordinate system, there are residual shifts between the confocal and MINFLUX coordinate systems that needs to be considered for optimal overlapping of confocal and MINFLUX data. The shifts depend on the scanning speed of the confocal image, as we can approximate the galvanometric mirrors as stationary during the MINFLUX acquisition, and the relative position in the applied scanning range of the galvanometric mirrors. Characterization and calibration of shift curves depending on scanning speed and position has here been performed where necessary, and shifts of up to 100 nm are applied to the data where necessary. This is not applicable only to etMINFLUX data, but any confocal and MINFLUX data recorded on the Abberior MINFLUX microscope. The shifts we have characterized depend on the scanning speed of the confocal image, where we can approximate the galvanometric mirrors to be stationary during MINFLUX acquisition.

The shift for a specific point in the image depends also on the distance from the start of a scanning line. The shifts occur both in the X and Y direction, with significantly different types of shift visible. While we have not understood the underlying sources of the full range of shifts we observe, we have fitted compensation curves to test measurements allowing us to correct for it in post-processing of data. Optimally, a slower confocal scanning is used, in which case no significant shift is observed and a compensation can be left out. Based on our testing, we have deemed a confocal linear scanning speed of 7 μm/ms (pixel size 70 nm, pixel dwell time 10 μs) safe to avoid causing shifts in need of compensation in our final data. In the Caveolin1 data, we have compensated for the shifts present, while in the Dynamin1 and Gag data we have used a slower confocal scanning speed.

The scanning-dependent coordinate shifts were measured by using confocal imaging with two different scanning speeds – very slow, to mimic a pseudo-stationary state as used during MINFLUX acquisition, and the scanning speed of choice in the experiments. The very slow speed was set to 0.7 nm/μs. With these settings, two confocal images were taken subsequently of a test sample containing fluorescent beads. Each bead position in the image was automatically detected in the two images, the beads found in both images were connected, and the position of them were fitted to subpixel precision using 2D gaussian fitting. The center of each peak was extracted, and the shift in X, fast scanning axis, and Y, slow scanning axis, for each bead in the two images was calculated. This shift can be assumed to be identical to the shift that would occur in a MINFLUX acquisition following a fast confocal acquisition, which was confirmed by compensating the shift in confocal-MINFLUX measurements of fluorescent beads with the shifts extracted from confocal-confocal measurements. The shift population can be plotted in a 2D scatter plot, where each datapoint is an individual bead, the horizontal axis shows the distance from the left border of the image for that bead, and the vertical axis shows the shift in either X or Y. Doing this, the spatially dependent shifts become apparent (Supplementary Figure 3). Recording multiple instances of pairs of images, across different days, confirmed the consistent spatially-dependent shapes of the shifts. In the figure, data from multiple image pairs has been overlaid and fitted together.

The X-shift of a non-bidirectional scan with a scanning speed of 35 nm/μs (Supplementary Figure 3a) takes the form of an initial second-degree polynomial followed by a sigmoidal function, while the Y-shift (Supplementary Figure 3b) takes the form of an oscillating sinusoidal function with an overall downwards slope. In a bidirectional scan with the same scanning speed, the X-shift (Supplementary Figure 3c) takes the shape of a linearly increasing function, while the Y-shift (Supplementary Figure 3d) takes the shape of a linearly decreasing function. Finally, the shifts from a bidirectional scan with a different scanning speed (50 nm/μs) are shown (Supplementary Figure 3e–f), confirming that the shape stays the same but the slope changes for different scanning speeds. This is also valid for non-bidirectional scans.

The large range of shift values measured for a specific position from the leftmost part of the image can be explained by random scan-to-scan shifts. The shifts are persistent between individual scans, can occur during or between frames, and appear as discrete jumps. As such, individual measurements show up as different bands in the plots of scan shift vs position along a certain axis. Individual measurements can also appear as multiple split bands, if these shifts happen to occur during the recording of a frame. As these shifts are randomly occurring, and

do not depend on acquisition parameters, they cannot be compensated for and result in a residual random shift.

Overall, the average scan shifts measured, which is what the compensation is based on, can be up to 150 nm large, depending on the scanning speed. The residual random and uncorrectable shifts can be determined from the plots to not be larger than ~50 nm, and hence is the maximum residual error after correction. Knowing this, a slower scanning speed to avoid the shifts is preferable to compensating for the shifts in post-processing of the data.

Supplementary Note 4. Napari widget for etMINFLUX data visualization

In order to enable easy visualization of the resulting datasets from etMINFLUX acquisitions, we have developed a napari widget that allows loading and visualization of recorded datasets from etMINFLUX experiments of any experiment mode available. This napari widget is available on GitHub (https://github.com/jonatanalvelid/napari-etminflux-data-viewer), as a release on Zenodo (https://doi.org/10.5281/zenodo.18712437), as well as directly to install from pypi to a local Python environment containing napari (https://pypi.org/project/napari-etminflux-data-viewer/). Note that the napari widget requires extraction of MINFLUX datasets to .npy format from Imspector, keeping the MINFLUX dataset names that etMINFLUX generates.

After installation, load the widget from the Plugins dropdown menu in napari. In the widget, a folder with experimental data can be loaded. The widget looks for .msr Imspector files, .tif confocal data files, .txt log files, and .npy dataset files. It will list all the events it found in the folder in a dropdown menu, from which a specific event to load can be selected. Before loading the selected event, make sure the settings match that during the recording of the etMINFLUX experiment. This includes the recording mode used in etMINFLUX, the Imspector version used for the experiments, the data dimensions (2D or 3D), and the sequence iteration index (zero-indexed) for the final localization in the sequence used. The latter needs to be defined manually, as any sequence can be used in etMINFLUX, but the default values for 2D and 3D tracking is 3 and 4 respectively.

Upon loading the data, all ROI MINFLUX data of the event (single or multiple ROIs, single or multiple timepoints) are loaded on top of the confocal data. Both confocal and MINFLUX data are loaded along the same common timeline, and all MINFLUX timelapse data are loaded in a concatenated way. The temporal axis can be scrolled through using the slider labelled time, in case of timelapse data. The temporal axis unit is in seconds from the timepoint of the MINFLUX data acquisitions initiation. Any confocal frames before that are, due to napari multi-dimensional stack limitations for scale and offset not allowing different intra-stack frame times or negative temporal axis offsets, present for the same amount of time as the latter confocal frames during MINFLUX acquisitions. In order to compensate for this, the user can add an overlaid text label that shows the real time, as well as two additional optional labels (confocal and MINFLUX labelling for example). In order for the time stamps to be correct, the user has to provide the time between confocal frames in the initial confocal-only monitoring step in the corresponding GUI field before selectin “Add overview label”. Additionally, the event

metadata as saved in the log .txt file is loaded and presented in a text box in the widget upon loading an event.

Supplementary Note 5. Real-time analysis pipelines

The etMINFLUX widget uses real-time analysis pipelines that shares a common structure, which allows them to be flexibly loaded in the widget, and allows users to develop new analysis pipelines that can be directly used for experiments. The pipelines are developed as Python functions with a specific initial call syntax, including the stack of previously recorded images, a potential binary mask, a fed-back Python object of any type, and an end call syntax that contains all the pipeline parameters that the user can tweak in real-time in the etMINFLUX main widget after loading the pipeline.

Three different real-time analysis pipelines were used during the process of this work, each one optimized at the task at hand in the three different application cases presented. While the pipelines share common features, it is important to note that each pipeline should be optimized to the specific sample at hand. The below-presented pipelines have each been optimized, methodologically and for parameter values, on training data recorded as confocal timelapses without etMINFLUX activated. These timelapses have been manually annotated as a ground truth for the events one would wish to detect, to which the outcome of the real-time analysis pipelines has been compared and further optimized. Each of the three pipelines are presented in detail below.

Real-time peak detection analysis pipeline

The peak_detection_bright real-time analysis pipeline was used in testing and characterization, in order to detect fluorescent beads, as well as in detection Caveolin1-EGFP accumulation sites, i.e. caveolae. The pipeline is supplemented to the etMINFLUX control widget as a separate Python module containing only the pipeline function. It uses only the last confocal image as input, and outputs the coordinates of any detected peaks, according to the pipeline parameters provided by the user. The pipeline has default values for all input parameters, and the full parameter value ranges that were used for the data recording in this work are presented in Supplementary Table 1. The pipeline uses the numpy[5], scipy[6,7], scikit-image[8], and opencv[9] packages. The pipeline is available on GitHub:
https://github.com/jonatanalvelid/etMINFLUX/blob/main/analysis_pipelines/peak_detection_bright.py.

The pipeline starts with a smoothing with a Gaussian filter, using the user-provider provided smoothing radius. The pipeline can take a binary mask as input, which if provided is used to mask the image after smoothing. Following this, the image is dilated, with a rectangular kernel of a user-provided size. The dilated image is compared to the original smoothed image, and any pixels with an equal value in the two images, and a peak value above a user-provided threshold, are considered peaks. The peaks are sorted according to pixel intensity, and any peaks inside a border limit of a user-provided size are removed. Furthermore, peaks that are too close to each other are removed, as they will be considered to be in a too crowded area. The distance between peaks that are considered too close is user provided. In the end, only the first N peaks in the list are returned, allowing the user to choose how many peaks at most they want.

Following the peak detection, if the user has requested local ROI sizes to be calculated, a further part of the analysis pipeline will take place. This part looks at the peak coordinates, and for each peak it gets a mask of the immediate surrounding of all pixels above a certain user-provided percentage of the peak value. From the mask, the x and y sizes of the smallest bounding box of the largest area in the mask will be calculated. These will be returned as the ROI size.

Multiple version of the pipeline exists, where the returned peak coordinates are sorted or filtered in a different way depending on the need: (1) brightest peaks first, (2) dimmest peaks first, (3) only the peak of a random peak index, or (4) only the peak of a user-provided peak index.

Dynamin rising signal analysis pipeline

The dyn_signalrise real-time analysis pipeline was used to detect dynamin1-EGFP rising over time on the seconds timescale, indicating an accumulation of dynamin1 and an endocytosis scission site. The pipeline is supplemented to the etMINFLUX control widget as a separate Python module containing only the pipeline function. It uses the last confocal image as well as a stack of the previously recorded frames as input, and outputs the final coordinates of any spot that has been considered as having a rising signal according to the conditions and pipeline parameters explained below. The pipeline has default values for all input parameters, and the full parameter value ranges that were used for the data recording in this work are presented in Supplementary Table 1. The pipeline uses the numpy[5], scipy[6,7], opencv[9], pandas[10], and trackpy[11] packages. The pipeline is available on GitHub: https://github.com/jonatanalvelid/etMINFLUX/blob/main/analysis_pipelines/dyn_signalrise.py.

The pipeline starts with a smoothing with a Gaussian filter of a fixed radius of 1.5 pixels. The image is then fed through two separate gaussian filters, with a 0.05- and 3-pixel radius respectively, and a difference of Gaussians image is calculated between the two. This step is in order to make the signal peaks clearer and more separated from spread-out background and noise in the image. Negative pixel values are at this point clipped and set to 0. Following this, a third step of Gaussian filtering is performed, once again with a 1.5-pixel radius.

After this, a peak detection is performed in the following steps: firstly, a rectangular kernel is determined using a user-inputted parameter as the size; secondly, the image is dilated with this kernel; thirdly, a mask containing the pixels that are equal in the dilated image and the image before dilation is calculated. Two thresholds, one high and one low, provided by the user are used to filter the mask based on the pixel intensities in the image prior to dilation of the peaks that are represented in the mask. This removes any detected peaks not inside the intensity range of interest. The coordinates of the peaks are put in a list and are sorted in a descending order according to their pixel intensities. The list is also cut after a number of peaks corresponding to a user-inputted parameter, for control over how many peak tracks to follow. The intensity summed in a 5-pixel-wide rectangle around each detected peak is extracted, and the timepoint, coordinates, and intensities are all put into a pandas dataframe. This dataframe is concatenated with the information from all previously recorded frames and runs of the pipeline, which is returned and inputted to each pipeline run.

After this, the conditional checking of tracks is initiated, to find the spots that increases in intensity. First, the dataframe is provided to the trackpy method link, that performs a single-

particle track linking algorithm and finds the most likely connected tracks from the peaks detected in each frame, using user-inputted parameters for the search range in time and space. After this, five checks are performed on each track, and an event is detected only if it passes all conditional checks. Check 1 checks if a track appeared, i.e. had the first timepoint of the track in the dataframe a certain number of frames ago, where the number of frames is provided by the user. Check 2 checks that the track stayed for most frames after that, and only allows it to be gone for one frame. Check 3 checks that the intensity of the track increase over time from appearance to the current frame. It does this by getting the mean of the intensity before appearance at the spot of appearance, the intensity track around appearance, and the intensity track after appearance, where each window is a few frames long, controlled by user-inputted parameters. The means are then turned into two intensity ratios, comparing the around appearance with before appearance, and the after with around appearance. Both of these ratios have to be inside a threshold range, controlled by two inputted parameter values, to be considered valid – above a threshold to actually be a true increasing signal and below a threshold to avoid detecting rapidly appearing signal that likely indicates a lot of movement or noise. Check 4 checks that the track has not moved too much since appearance, and does so by taking the mean Euclidean distance the spot has moved between frames since appearance, and compares this mean to a user-provided threshold. This again ensures a decrease in false detections as the dynamin should accumulate at a relatively fixed point on the membrane. Finally, check 5 checks that the last position of the track is not at the border of the image, with the border size decided by a user-inputted parameter, as this otherwise hinders the MINFLUX ROI from being set correctly. If all conditional checks are passed, the event coordinates are extracted and the pipeline is immediately returned, as we are not interested in any potential additional sites as the endocytosis happens too fast to be able to catch multiple scission events simultaneously.

Gag rising signal analysis pipeline

The gag_signalrise real-time analysis pipeline was used to detect Gag-EGFP rising over time on the minutes timescale, indicating an accumulation of Gag and a potential virus budding site. The pipeline is supplemented to the etMINFLUX control widget as a separate Python module containing only the pipeline function. It uses the last confocal image as well as a stack of the previously recorded frames as input, and outputs the final coordinates of any spot that has been considered as having a rising signal according to the conditions and pipeline parameters explained below. The pipeline has default values for all input parameters, and the full parameter value ranges that were used for the data recording in this work are presented in Supplementary Table 1. The pipeline uses the numpy[5], scipy[6,7], opencv[9], pandas[10], and trackpy[11] packages. The pipeline is available on GitHub: https://github.com/jonatanalvelid/etMINFLUX/blob/main/analysis_pipelines/gag_signalrise.py.

The image preprocessing starts with a Gaussian smoothing step with a 1.5-pixel radius kernel, followed by a peak detection similar to the peak detection described for the dynamin-detecting pipeline: dilation with a rectangular kernel, comparison between dilated and before-dilation images, and extraction of pixels with an equal intensity. The peak coordinates are filtered with a high and low threshold, sorted according to intensity, and everything beyond a certain number

of peaks is removed. After this, a pandas dataframe with the time, coordinates, and intensity in 5-pixel-wide rectangle is defined, and added onto any previous data that is refed into the pipeline with every run.

After this, the conditional checking of tracks is initiated, to find the spots that increases in intensity. First, the dataframe is provided to the trackpy method link, that performs a single-particle track linking algorithm and finds the most likely connected tracks from the peaks detected in each frame, using user-inputted parameters for the search range in time and space. After this, seven checks are performed on each track, and an event is detected only if it passes all conditional checks. Check 1 checks if a track appeared inside 1–3×frames_appear ago, where frames_appear is a user-inputted parameter – here a range of frames are considered as it increases the chance that an event does not go undetected due to noisy intensity traces. Check 2 checks that the peak stays detected for at least 70% of the frames following the appearance, allowing some flickering in peak detection during the early stages of the site where the intensity is close to the threshold. Check 3 checks if the distance to all other peaks during appearance is above a user-provided threshold, which ensures that no events are detected due to the tracks of two or more peaks crosses each other and the become visible as multiple peaks again. Check 4 checks that the intensity of the peak increases over time. This check is here performed differently depending on the length of the frames available and number of frames since the track appeared. If there are not enough frames available, similar intensity ratio checks between before, during, and after appearance as described for the Dynamin1 detection above are performed, while if more information is available, a curve fitting takes place to be more precise and robust to noise. A linear function is fitted to the track intensity over time, with a fixed intensity at t=0, and the slope of the fit is extracted. If the slope is above a user-provided threshold (usually between 4–10%/frame), or in the previous case if the intensity ratios are passed, check 5 is performed. This check checks that the final intensity is above a lower threshold and below an upper threshold, to avoid noisy, low-intensity detections and likely other high-intensity false detections. Check 6 looks at the mean moving distance between frames for all frames where the peak is detected, and compares this to an upper threshold. Finally, check 7 checks that the last position of the track is not at the border of the image, with the border size decided by a user-inputted parameter, as this otherwise hinders the MINFLUX ROI from being set correctly. If all conditional checks are passed, the event coordinates are extracted and the pipeline is returned.

Supplementary Note 6. Analysis pipeline parameter optimization and performance evaluation

In order to perform parameter optimization and performance evaluation on the analysis pipelines, we implemented an automated hyperparameter tuning routine. While we keep the actual structure of the analysis pipeline fixed, this allows us to both optimize the parameters to specific timelapses, as these values will vary with the exact acquisition settings and sample conditions, and evaluate its performance. Taken as the resulting range of parameter values over different timelapses and samples, the results from the performance evaluation can be seen as an upper limit benchmark as of how well we can expect the analysis pipeline structure to optimally perform. In the end, this evaluation and parameter optimization closely matched that which we

found in the etMINFLUX experiments. It is important to note that with slightly different acquisition settings or pipeline parameters, the pipeline might perform better or worse, and constant parameter checking and tweaking is needed during experiments. This is also the reason as to why the optimization results show a higher precision as compared to the real etMINFLUX experiments, where parameter tweaking has been adjusted on the fly by an expert user.

The optimization and evaluation routine were performed by treating the analysis pipeline as a black-box model with a set of tunable parameters. We used annotated confocal timelapses as ground truth and evaluated a parameter value set by running the pipeline on a timelapse and matched predicted events to annotated events with a certain spatial and temporal tolerance (Dynamin1: dt = 40 frames, dr = 6 pixels; Gag: dt = 30 frames, dr = 6 pixels), leading to a set of true positives, false positives, and false negatives, and in the end a recall, precision, and $F\beta$ score. The parameter value set was optimized between runs using Bayesian optimization from a range of values for each parameter to optimize the $F\beta$ score. In the end, after 100 trials, a best parameter combination was found that maximized the $F\beta$ score. We used different β values for Dynamin1 and Gag pipeline optimizations, premiering either precision for the Dynamin1 case (β = 0.25), thanks to the abundance of events, and recall for the Gag case (β = 4), due to the lack of events and possibility to scrutinize the event detections manually before entering the ROI following loop. We ran the optimization on each timelapse separately, in order to find an upper bound of what we can expect as a pipeline performance.

For the Caveolin1 peak detection pipeline, an optimization routine would essentially only handle three parameters: absolute threshold, smoothing radius, and minimum distance between peaks. As these parameters do not affect each other noteworthy, and the pipeline is simplistic in its nature (see Supplementary Note 5), it is easy for even an uninitiated user to tweak the pipeline to detect the peaks of interest. As the outcome of this pipeline is extremely dependent on the intensity thresholding (for example, if one wants to only trigger events at bright peaks, the threshold is set high), and how many peaks one wants to run the following analysis on, comparing parameter optimization routine runs on different datasets where there are no clear rules as to what peaks to annotate would not render comparable parameter values. Instead, we decided an intensity threshold (~50, high, to only look at the bright, more stationary peaks) and peak distance (~10 pixels, medium, to only look at singled-out peaks with less noise around them) on a single confocal frame, and an expert user annotated the peaks to detect in that frame. The parameter optimization routine was then run on the frame, and the results are the following: the absolute threshold was optimized to 48.8, the smoothing threshold was optimized to 1.1 pixels, and the minimum distance between peaks was optimized to 14.6 pixels. In the end, this generated 22 true positives, 0 false positives, and 8 false negatives, for a $F\beta$ score of 0.98 with a β value of 0.25 to favor precision (= 1.0) and thus minimize MINFLUX acquisitions at unwanted peaks. The recall was 0.73.

For the Dynamin1 pipeline, the resulting optimized parameters from the parameter optimization closely matched the values used during etMINFLUX experiments, as reported in Supplementary Table 1, indicating well-optimized pipeline running close to optimal performance on the available sample conditions. After running the pipeline on 8 individual confocal timelapses, from 2 different samples, each of 100–300 frames, the range of optimized

parameter values were the following (other parameters were kept fixed, assumed to not affect the outcome):

min_dist: [1.0, 1.9], thresh_abs_lo: [0.55, 1.25], thresh_abs_hi: [10, 32], memory_frames: [3, 6], track_search_dist: [5, 7], frames_appear: [6, 10], thresh_intincratio: [1.1, 1.28], thresh_intincratio_max: [3.8, 4.3], thresh_move_dist: [1.0, 1.47]

The resulting recall, precision, and $F\beta$ values are presented in Supplementary Figure 18a. The average precision is 1.0, with the recall at 0.18. The mean $F\beta$ value after optimization is 0.72.

For the Gag pipeline, the resulting optimized parameters from the parameter optimization routine also closely matched those used during etMINFLUX experiments, as reported in Supplementary Table 1. After running the pipeline on 3 individual confocal timelapses divided into 12 individual tiles, from 3 different samples, each of 100 frames, the range of optimized parameter values were the following (other parameters were kept fixed, assumed to not affect the outcome):

min_dist_appear: [1.2, 6.5], thresh_abs_lo: [1.1, 6.0], thresh_abs_hi: [10, 59], finalintlo: [0.65, 1.15], finalinthi: [31, 66], memory_frames: [2, 5], track_search_dist: [2.2, 4.9], frames_appear: [3, 8], thresh_intincratio: [1.05, 1.49], thresh_intincratio_max: [7.5, 29], intincslope: [0.040, 0.13], thresh_move_dist: [0.74, 1.99]

The resulting TP, FP, FN, and Fβ values are presented in Supplementary Figure 18b. The average precision is 0.45, with the recall at 0.63. The mean Fβ value after optimization is 0.67. These values indicate the difficulty in detecting the slow and dim signal accumulation of gag.

Supplementary Note 7. Data throughput comparison between etMINFLUX and manual acquisition control

In order to compare etMINFLUX with manual acquisition control, we mimicked the etMINFLUX experiments with manual microscope control in the three application areas. In doing this, we could compare both the feasibility and data throughput aspects.

For the application on caveolae, we performed manual acquisitions of ~10 ROIs per cell by always selecting a Caveolin1 peak as the ROI center, while keeping ROI size and other MINFLUX acquisition parameters the same as with etMINFLUX. From this, we could measure the total experiment time. We calculated the same times for etMINFLUX experiments. To accurately compare experiments with different number of MINFLUX ROIs and different ROI dwell times, we calculated the extra time spent per ROI in terms of dwell times, i.e. the overhead factor. This number gives an idea of how much time is spent between acquisitions on other actions such as running confocal images, selecting ROIs, data handling, and data saving. EtMINFLUX has an average overhead factor of 1.16, with a very small spread, while manual control has an average overhead factor of 1.49 and a larger spread (Figure 3f). Comparatively, this means that the temporal losses between MINFLUX acquisitions is cut by a factor of ~3× by using etMINFLUX, leaving the extra time per ROI at ~10 s vs ~30 s (Supplementary Figure 11).

For the application on endocytic vesicles, we wanted to compare both the temporal as well as useful-data throughput. Similar to above, we performed manual control acquisitions, attempting to detect Dynamin1 events and performing MINFLUX at the event sites just as with etMINFLUX. Here, due to the more complex and rare nature of the event, the biggest time loss is simply looking for the next event. We measured the total experiment time or a number of events or ROI cycles, and subtracted the actual time spent on MINFLUX acquisitions. With this, we get the time spent looking for the next event, averaging 109 s in manual experiments. Using etMINFLUX, this went down to 50 s and occasionally being as small as 10 s, overall giving an improvement factor of 2× (Figure 4e).

Analyzing instead the useful data throughput, i.e. valid events with endocytic vesicles present overlapping the Dynamin1 signal, we found that the efficiency at which we can catch the vesicles was roughly 26% using etMINFLUX. With manual control, the same figure was 9%, indicating the difficulty in actually catching the vesicles in time before they are scissioned. This means that etMINFLUX is 3× better at catching useful data, and in total with the temporal improvement above, the overall improvement in data throughput for second-scale cellular processes using etMINFLUX is ~6×.

For Gag, we attempted manual acquisitions in the same way. In the end, only a single event was captured, and it did not show any bulging or budding present despite following it for 35 min (Supplementary Figure 24). In these types of processes, the huge benefit of using etMINFLUX is clear. Even if events were captured at a higher rate, the difficulty of temporally aligning them would mean that many types of downstream analysis would be hindered.

Supplementary Note 8. MINFLUX track data filtering steps in caveolae analysis

Single-molecule lipid tracks from MINFLUX data were filtered before lipid diffusion analysis took place. Tracks were filtered on a track basis, where for each track three quality checks were performed. The first check was to see that the temporal track length was longer than 30 ms, in order to ensure enough statistics for better diffusion coefficient estimates in the downstream analysis. The other two checks were made to ensure that the track was not just depicting a fluorophore seemingly stuck in the same place. Depending on the probe used, we observed more or less stuck tracks, and while the source of these stuck tracks is unknown it is beyond the scope of this study to further understand them. We focused on the tracks that showed movement away from such a stuck position at some point during the track. In order to sort out these tracks, we checked that the mean distance moved between positions with a 50 localizations interval along the full track was above 13 nm, and that the mean of the standard deviations of the positions from a 40-localizations-long sliding window was above 20 nm. These thresholds were set by judging the filtering applied to test datasets, tweaking the threshold values until a balance between filtering faulty tracks and keeping diffusing and long enough tracks was found. An example of the filtering can be found in Extended Data Figure 3, where blue indicates short tracks, gray indicates a failed mean movement distance threshold, red indicates a failed mean standard deviation of positions threshold, and green indicates that all checks were passed.

Additionally, the confocal data of the caveolae sites was used in order to ensure that the caveola at which the diffusion analysis was going to take place did not move significantly during the MINFLUX recording wherever data was available to support this analysis. In order to do this, the confocal frames before the MINFLUX acquisition, as well as after the MINFLUX acquisition in terms of the confocal image before the subsequent MINFLUX acquisition if performed in the same FOV, were extracted. Small ROIs around the detected caveolae were extracted and a 2D Gaussian function was fitted to the intensity signal. The center of the fitted Gaussian was compared before and after the MINFLUX acquisition, and if the distance between the two centers was larger than 70 nm, i.e. roughly one pixel in the confocal images, the peak was considered to have moved and no diffusion analysis was performed. In the datasets were the confocal data necessary was not available, higher intensity cav1 peaks were selected which were considered from confocal timelapses to be more stable. The fitted caveola position in the confocal frame before the MINFLUX acquisition was used as the center position of the caveola in subsequent spatial analysis.

Supplementary Note 9. Diffusion analysis on 2D and 3D MINFLUX tracking data

In MINFLUX tracking data, the time steps (dt) between localizations are generally not equal due to various factors but mainly three; the target coordinate pattern can repeat due to not reaching the photon limit and will thus multiply the time step with an integer factor, there is an additional time due to calculations and preparations for the next localization, and there is temporal jitter. Together, this means that time steps are uneven and thus complicates the use of standard mean squared displacement (MSD) analysis as used in single-particle tracking with a camera, where the time steps are defined as the frame interval. Several approaches can be taken to ensure an accurate diffusion analysis from the MINFLUX data, and the one we employ here is a population-based square displacement analysis.

To perform this analysis, we start by gathering all possible time steps and their corresponding particle displacement distances (dd) between localizations up to a time step limit from the track or population of tracks we want to extract a diffusion coefficient for. Whereas an MSD analysis at this point would take the mean displacement for all displacements of a certain time step, and then fit a function to the means, our square displacement analysis instead fits a function directly to the 2D population of values on the form (dt, dd). The function fitted is identical to that which would be used in MSD analysis. From the fit, a diffusion coefficient can be extracted, if the number of displacements on which the fit is performed exceeds a certain threshold to ensure a reliable value.

The fitting function used in this work is the following function previously introduced by Michalet and Berglund[12] and further refined by Balzarotti et al.[13] for MINFLUX analysis:

$$dd = 2dDdt + 2d\sigma^2 - 4dR_{blur}Ddt_{median},$$

where dd is the displacement, dt is the time step, d is the number of dimensions of the diffusion model, D is the diffusion coefficient, sigma is the dynamic localization precision, R_{blur} is the motion blue coefficient, and dt_{median} is the median value of the time step population. In the diffusion analysis performed here, R_{blur} was set to 1/6.2 [13] and d was set to 2, as the diffusion

model assumed was always two-dimensional on a membrane even when the tracking performed was in 3D. dt_{median} was set to 85.7e-6 and 350e-6 in the 2D and 3D tracking analysis, respectively.

In our analysis, we extract both diffusion coefficients and transient diffusion coefficients. The difference between these is only the population on which we perform the analysis. For diffusion coefficients, we perform the analysis on the displacement population of a full track or the sections of the track that are inside respectively outside the defined radius of the event site. For transient diffusion coefficients, we perform the analysis on the displacement population of a sliding window around the localization to which we assign the fitted diffusion coefficient.

A main limitation of the diffusion coefficient analysis applied on cases where we want to calculate the diffusion coefficient in a small site area is the bias that is introduced due to the limited area. As we require a certain number of displacements for a fit to be reliable, for a small enough radius the only track sections that will meet that requirement are those that linger for a longer time at the site, and not those that pass it faster. Thus, the diffusion coefficients inside a site will be biased towards lower diffusion coefficients. Analysis performed on simulated diffusion data (Supplementary Note 10), with a diffusion coefficient and localization uncertainty modelled after that observed in our MINFLUX tracking data, indicated that a 200 nm radius was necessary to avoid such bias, and hence this radius was used for the analysis (Supplementary Figure 13). The transient diffusion coefficient analysis does not suffer from such bias, and hence a smaller radius can be used.

Supplementary Note 10. Simulated Brownian motion and MINFLUX tracking

The simulated MINFLUX tracking data was simulated as a Brownian motion process. For each set of parameters, 10 ROIs each with 100 tracks were simulated. The track length (in number of localizations) was determined individually for each track by taking a random value from an exponential distribution with scale parameter 250, matching the distribution of the real MINFLUX 2D tracking data. For each new localization in the iterative track simulation, a random time step dt is first pulled from a weighted discrete distribution matching that of real experimental data, to mimic the uneven time steps of MINFLUX data. Then, two values were added to the previous localization for each dimension: (1) a step with a random value pulled from a normal distribution with a standard deviation of $\sqrt{2Ddt}$, where D is the simulated diffusion coefficient and dt is the randomized time step, to simulate the molecule movement; and (2) a random value pulled from a normal distribution with a standard deviation equal to the extracted dynamic localization precision from the real MINFLUX tracking data, i.e. 9 nm, to mimic the localization process. To mimic the sites of Caveolin1 accumulation with a lower diffusion coefficient, the diffusion coefficient D is dependent on the molecule position. At each new localization step, the distance to the simulated site center is checked, and depending on if it is above or below the simulated site radius, here set to 50 nm, D_{in} or D_{out} is used.

To mimic the MINFLUX acquisition process and potential loss of the molecule during tracking due to the molecule moving too far from the targeted coordinate pattern center: if the molecule movement step is larger than 75 nm the molecule is considered lost and the track is terminated.

Supplementary Note 11. Geometrical analysis of endosomal vesicles

The endosomal vesicles before scission in the Dynamin1 accumulation sites data were geometrically analyzed using a pipeline centered around a convex hull fit. All parameters of the following filtering and analysis were optimized for each endosome, to ensure an accurate extraction of geometrical values. To start with, assuming a relatively flat membrane, which was the case in a vast majority of recorded data, the average membrane position was found using Gaussian fitting of the summed z-profile. Depending on if one or both membranes were visible in the tracking data a single or a double Gaussian function was used. The peak position was considered the membrane position, and the σ of the Gaussian was considered the membrane width. Following this, all localizations of the membrane were filtered out, and only localizations at a distance of generally 1–2σ above the membrane were kept. After this, a DBSCAN clustering algorithm[14] was used to cluster the data into clusters, with each cluster ideally representing an endosome. In most cases, only one cluster was found, while in occasional cases where multiple endosomes or other noisy tracks were present a label corresponding to the endosome in question was manually chosen. A minimum number of samples of 100 and an eps of 0.04 was generally chosen for the clustering algorithm.

With the localizations of the endosome cluster, an outlier removal was performed prior to the convex hull fitting, to get a more accurate shape of the endosome that otherwise would always be overestimated. The outlier removal used the IsolationForest function from the scikit-learn package[15], using 10 estimators and contamination at 0.25. Following this, a convex hull was fitted, using the ConvexHull function of the scipy package[6,7]. The surface area A and volume V were extracted from the fit, and the sphericity S was calculated as:

$$S = \frac{\pi^{1/3}(6V)^{2/3}}{A}.$$

The equivalent spherical diameter D was calculated as:

$$D = 2\left(\frac{3V}{4\pi}\right)^{1/3}.$$

Lastly, the neck length was calculated as the difference between membrane position in z and $D/2$ subtracted from the z-centroid of the convex hull.

For endosomal vesicles present in multiple MINFLUX acquisition cycles, the cycle with the clearest endosome visible in the data was chosen to be analyzed.

Supplementary Note 12. Z-artefact filtering of 3D MINFLUX tracking data

A common artefact in 3D MINFLUX tracking data are z-artefacts, where the track artificially continuously seems to move far in z in the same direction localization after localization, despite this being unreasonable due to the sample constrictions (such as these tails entering into the cover glass), while having longer times between localizations, due to pattern repeats. They occur more in data with a higher fluorophore concentration, and are therefore likely connected to background signals affecting the MINFLUX localization process, further corroborated by the

fact that multiple pattern repeats are needed to meet the photon limit criteria. In order to allow visualization and accurate analysis of the data, such artefacts are filtered away in any 3D MINFLUX data as a first step in the post-processing and analysis of the data.

The filtering is based on the longer time between localizations (dt) observed during the artefacts. It uses a sliding window approach, and calculates the average dt in a window of 70 localizations for each localization. For the sequences used in the experiments of this work, a dt threshold was set to 550 μs, and all localizations with a sliding window threshold above that was flagged as artefacts. The sliding window size and dt threshold need to be optimized depending on the sequence and excitation laser power used.

Supplementary Note 13. EtMINFLUX data handling

During an etMINFLUX experiment, the data is saved according to the following structure. (1) Up until an event detection, the raw confocal frames are saved in a timelapse in the _conf-raw.tif file, and any return processed images from the analysis pipelines, in the img_ana parameter, are saved in a timelapse in the _conf-analysisprocessed.tif file. The processed confocal images can contain any information that the user wants returned from an analysis pipeline, that can help in downstream analysis, and does in the pipelines presented in this work contains the initially processed image on which the peak detection in each of the three cases is performed. (2) When an event detection and therefore a MINFLUX acquisition takes place, the MINFLUX dataset is saved in Imspector with a name that contains information of the ROI number, pixel position in the confocal image, ROI size, and ROI recording time. (3) In ROI following modes, any confocal frames between MINFLUX acquisitions are saved in separate tiff files, again as _conf-raw.tif and _conf-analysisprocessed.tif. (4) Upon finishing an etMINFLUX experiment, whether it be after interrupting it or allowing it to naturally finish, two additional files are saved: the _log.txt log file, and the _minflux.msr Imspector data file. The log file contains metadata information from the etMINFLUX experiment, such as analysis pipeline parameters, timings, and other etMINFLUX settings. The .msr file contains all the MINFLUX datasets as well as the, potential, last confocal frame that in the case of ROI follow modes is recorded after the last MINFLUX acquisition has been performed.

Common for all files and MINFLUX datasets is that they are saved with a prefixed date and timestamp which is pulled at the moment that the data is initiated to record (MINFLUX datasets) or saved (all other files). For aborted etMINFLUX runs, when no even has been found, only the raw and analysis confocal frames are saved together with a log file.

In order to perform further visualization and analysis, we chose to extract individual MINFLUX datasets as .npy files. We provide a script to do this efficiently and automatically for all MINFLUX datasets in all .msr files in a folder: msrtonpy_folder.py. This script requires specpy and active Imspector connection.

The analysis scripts in the GitHub repository, https://github.com/jonatanalvelid/etMINFLUX-analysis-public, are then divided in different folders, and all scripts are presented in the form of Jupyter notebooks. All these scripts run on saved data and folder structures that are according to the way etMINFLUX saves them originally, plus the extracted .npy files according to above. This means that the folders should contain raw confocal data, .msr complete files, as well as

MINFLUX datasets in the .npy format. Example data for each experiment type are further provided in the folder for easy reference. The readme file in the repository further describes the different folders.

The confocal event detections can be analyzed and sorted through using the scripts in the event_inspection folder. These scripts plot the confocal event detections for the Dynamin1 and Gag accumulation events, mainly by saving PDF files for each detected event that plots zoomed-in individual confocal frames around the event detection, together with intensity plots with the summed intensity around the event detection coordinate in each frame. In both, the event frame and position are marked in green. With these plots, it is easy to sort correct event detections from eventual faulty ones, for events of interest that are similar intensity accumulation events. Faulty event detections from moving spots (non-membrane accumulations) or quickly appearing ones (moving in focus) can be seen from the events and intensity curves, despite having triggered the event detection due to the thresholds used.

The scan_shift scripts perform the confocal-MINFLUX scan shift calculations described in further detail in Supplementary Note 3. They do this either for all files in a single folder, or combined for multiple folders at the same time.

The metadata scripts extract metadata from the MINFLUX datasets, analysis parameters for the event detection log files, and runtimes for the analysis pipelines.

The cav1_analysis, dyn1_analysis, and gag_analysis scripts instead perform the specific analysis that are the basis for the results presented in this work. The specific important analysis types are described in further detail either in the Methods section or here as individual supplementary notes.

The pipeline_evaluation scripts perform the pipeline parameter optimization and performance evaluation on pre-recorded confocal timelapses, from which precision, recall, and fbeta was extracted.

DiffusionAnalysis and DiffusionAnalysis3D contains the classes used in the data handling of the lipid tracking data, in 2D or 3D respectively, mainly to perform diffusion analysis but also in general to handle the generated data. They take complete etMINFLUX data folders as input, loading the data into the class object, and allows to perform an array of different analysis.

Supplementary Figures

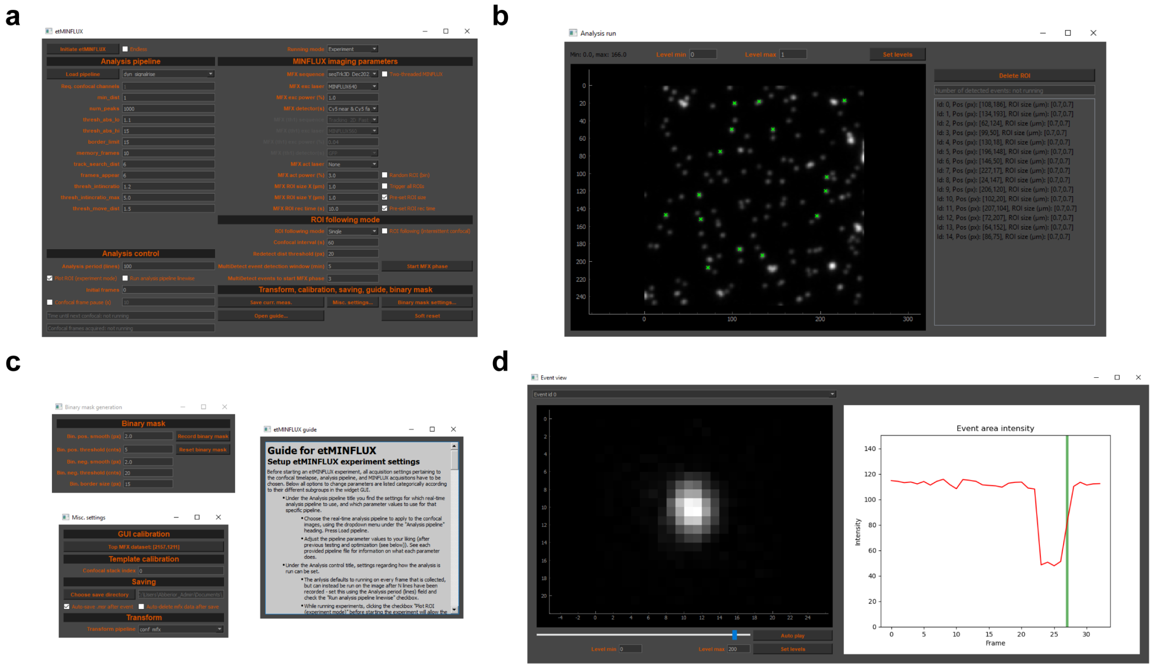

Supplementary Figure 1. etMINFLUX widget GUI. a. GUI of main widget window, with analysis pipeline settings (top left), analysis control (bottom left), coordinate transform, GUI calibration, and binary mask settings (top right), MINFLUX acquisition settings (middle right), and ROI follow mode and saving settings (bottom right). **b.** Analysis pipeline live-view and event detection sites window, with processed analysis image and overlaid detected events (left) and interactable list of detected events (right). **c.** Binary mask generation window (top left), miscellaneous settings window (bottom left), and guide window (right). **d.** Event view window, with a time lapse viewer of a zoom-in of the chosen detected event (left) and an intensity graph over the full time lapse of the immediate surrounding of the event site (right).

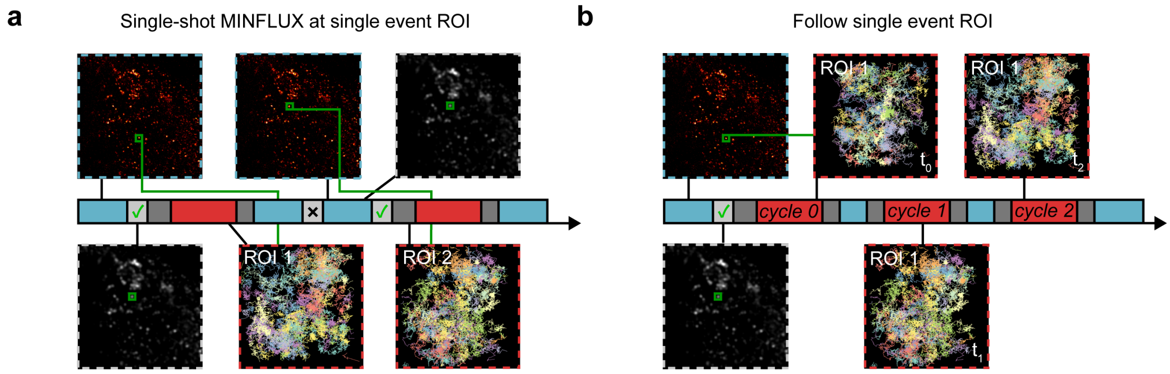

Supplementary Figure 2. Further experimental modalities enabled by event-triggered MINFLUX. a. Single event ROI recording after a single event. **b.** Single event ROI following, with interleaved confocal and MINFLUX recordings. Timescales show confocal acquisition (blue), MINFLUX acquisition (red), real-time analysis (light gray) and overhead (dark gray).

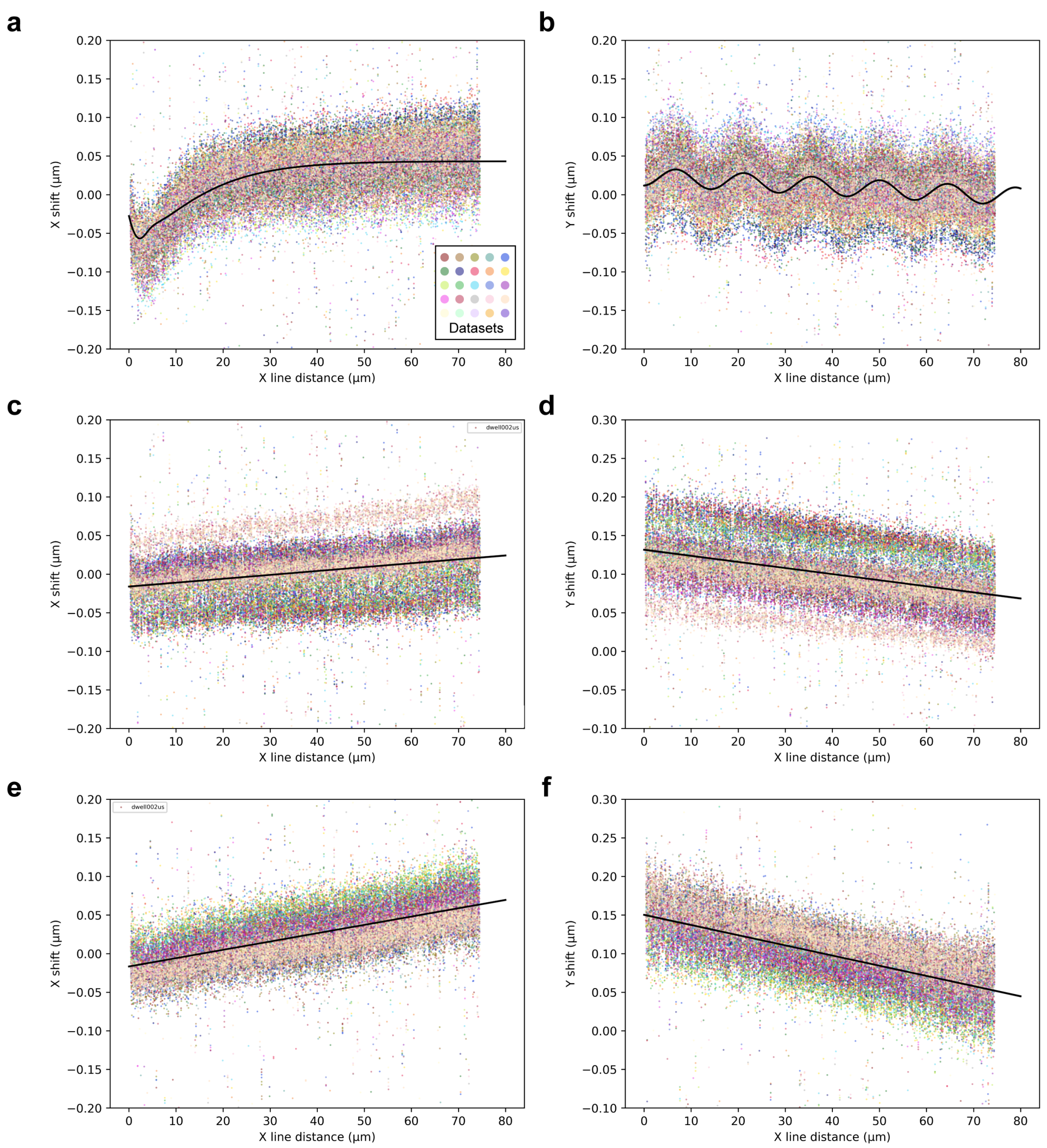

Supplementary Figure 3. Scanning-dependent confocal coordinate shifts. Acquisition parameter dependent coordinate shifts between fast and slow confocal image acquisitions. Each datapoint represents the measured X- or Y-axis shift (vertical axis) at the bead distance from the start of the fast-axis line (horizontal axis) for a fluorescent bead in a fast and slow image pair of the same sample region. The data from multiple image pairs have been overlapped, plotted with each dataset (N=25), i.e. image pair, in a unique color. The black line shows the mean fit and the black shaded area shows the 95% confidence interval of the fitted function. **a.** X-shift for unidirectional scanning with a scanning speed of 35 nm/µs. n=52610 beads. **b.** Y-shift for unidirectional scanning with a scanning speed of 35 nm/µs. n=52610 beads. **c.** X-shift for bidirectional scanning with a scanning speed of 35 nm/µs. n=52820 beads. **d.** Y-shift for bidirectional scanning with a scanning speed of 35 nm/µs. n=52820 beads. **e.** X-shift for bidirectional scanning with a scanning speed of 50 nm/µs. n=48860 beads. **f.** Y-shift for bidirectional scanning with a scanning speed of 50 nm/µs. n=48860 beads. **a–f.** All datasets individually come from n=25 images from n=5 independent experiments. Source data are provided as a Source Data file.

a

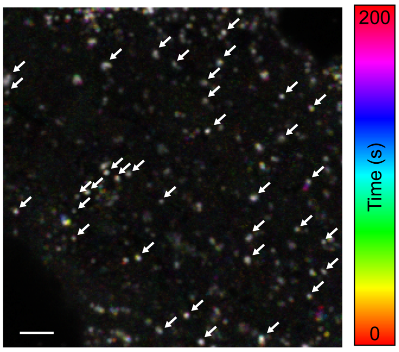

Supplementary Figure 4. Stability of Caveolin1 spots in a confocal timelapse. a. Timelapse confocal measurement with a frame interval of 2 s over a time period of 200 s shows the stability of caveolin spots over time, with exemplary stable spots marked with arrows. Representative example from n=5 experiment repetitions. Brighter spots were selected as event sites with etMINFLUX. Scale bar: 2 µm.

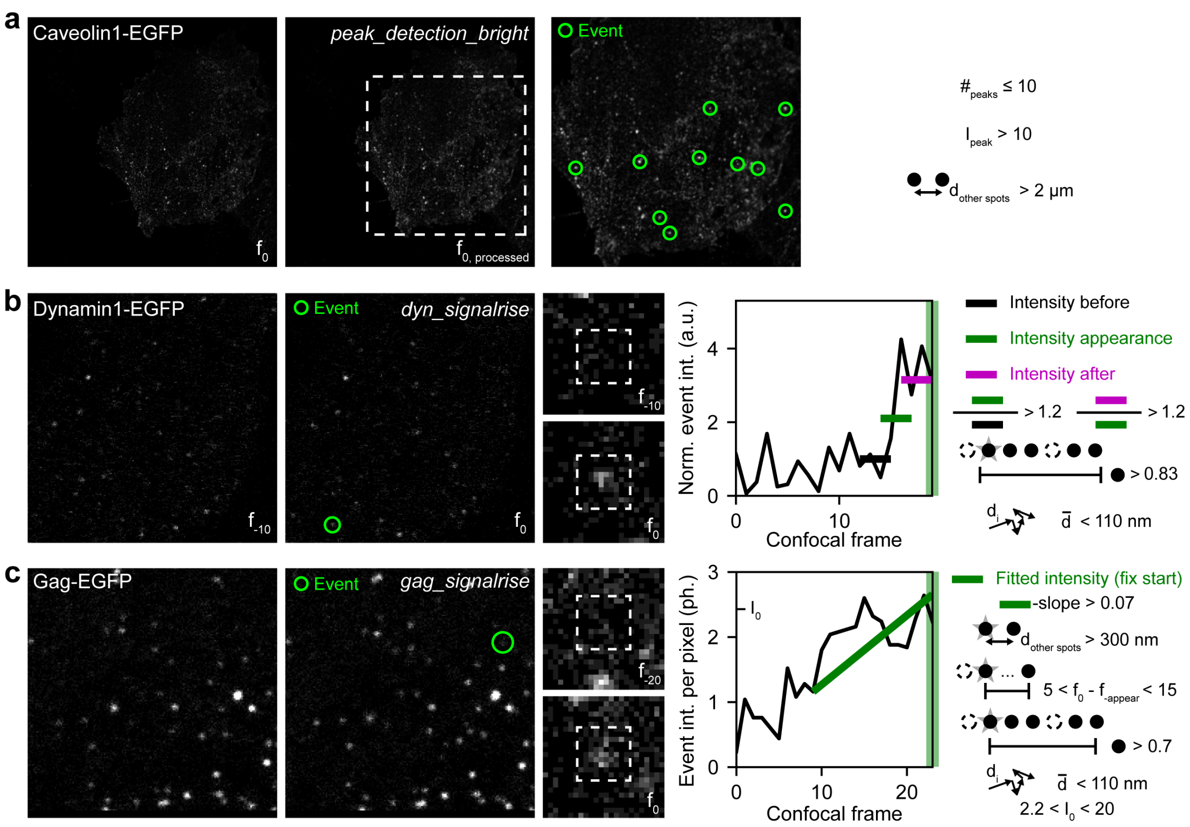

Supplementary Figure 5. Schematics of analysis pipelines. a. Schematic of peak_detection_bright used to detect Caveolin1-EGFP peaks, with raw confocal frame (far left), processed confocal frame (middle left), detected events (middle right), and conditional checks (far right). **b.** Schematic of dyn_signalrise used to detect rapid accumulation of Dynamin1-EGFP, with raw confocal frame -10 (far left), raw confocal frame 0 (middle left), zoom in to event area for the two confocal frames (middle), intensity curve at event site (middle right), and conditional checks for event detection (far right). In the intensity curve, the event frame is marked with a vertical green line, and three calculated intensities are marked with horizontal black, green, and magenta lines. Conditional checks are: (1) intensity ratios above threshold, (2) peak persistency after peak appearance, and (3) peak movement below threshold. **c.** Schematic of gag_signalrise used to detect slow accumulation of Gag-EGFP, with raw confocal frame -20 (far left), raw confocal frame 0 (middle left), zoom in to event area for the two confocal frames (middle), intensity curve at event site (middle right), and conditional checks for event detection (far right). In the intensity curve, the event frame is marked with a vertical green line, and the fitted intensity line with a fixed starting point in the intensity curve is marked as a sloped green line. Conditional checks are: (1) slope of fitted intensity trace with fixed initial intensity above threshold, (2) peak distance to other peaks, (3) peak appearance in a range of previous frames, (4) peak persistency after peak appearance, (5) peak movement below threshold, and (6) final intensity between thresholds.

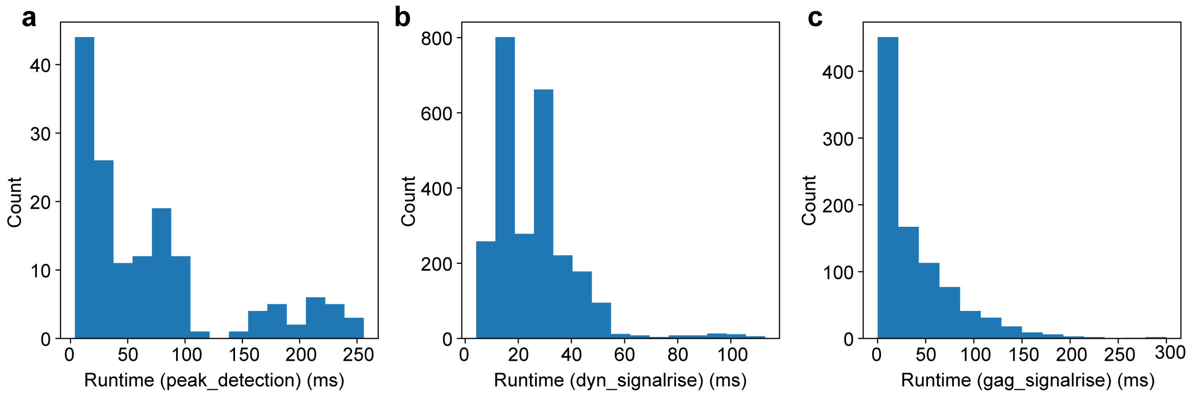

Supplementary Figure 6. Analysis pipeline performance. Mean (bar height) run time for analysis pipeline applied in the experiments, with one standard deviation (error bar) shown. **a.** Runtimes for peak_detection_bright analysis pipeline used in Caveolin1 caveolae site experiments. n=153 pipeline runs from n=4 independent experiments. **b.** Runtimes for dyn_signalrise analysis pipeline used in Dynamin1 endocytosis experiments. n=2563 pipeline runs from n=4 independent experiments. **c.** Runtimes for gag_signalrise analysis pipeline used in Gag accumulation experiments. n=709 pipeline runs from n=12 independent experiments. Source data are provided as a Source Data file.

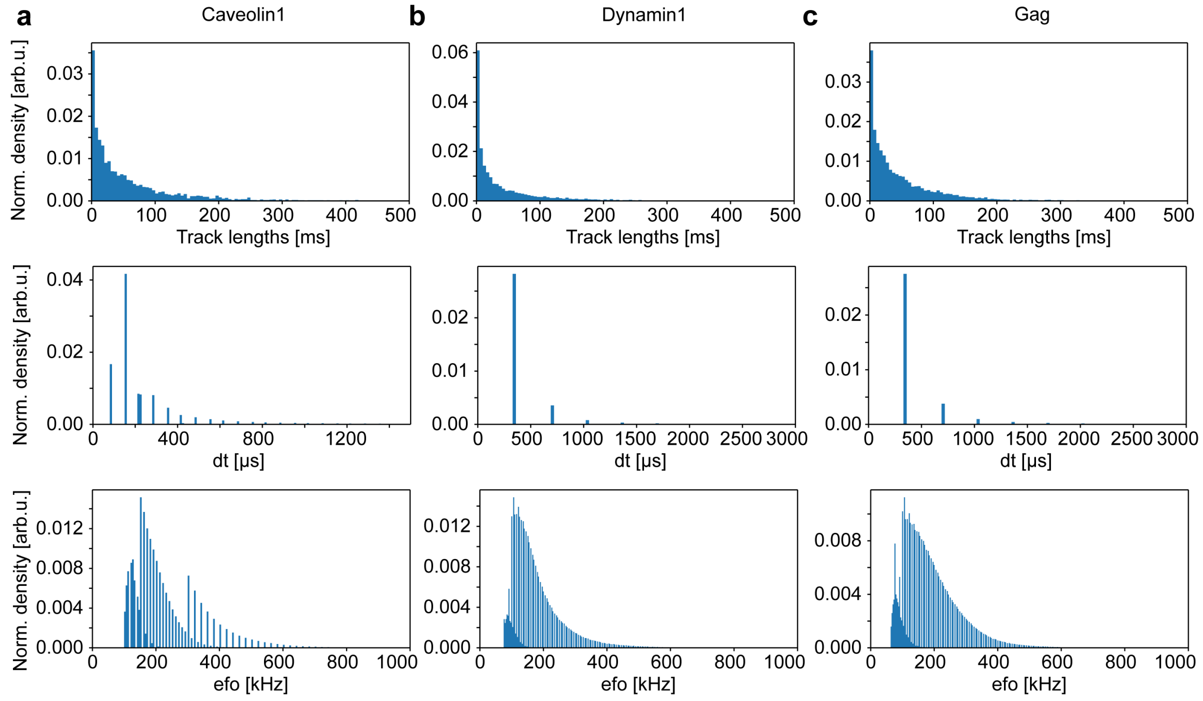

Supplementary Figure 7. Exemplary dataset metadata value distributions. Representative metadata value distributions for one representative dataset from each experiment type, showing track length, time between localizations, and efo distributions, for a Caveolin1 dataset in **a**, a Dynamin1 dataset in **b**, and a Gag dataset in **c**. Caveolin1: n=575611 localizations from n=3144 tracks; Dynamin1: n=578320 localizations from n=6036 tracks; Gag: n=414295 localizations from n=2617 tracks. Source data are provided as a Source Data file.

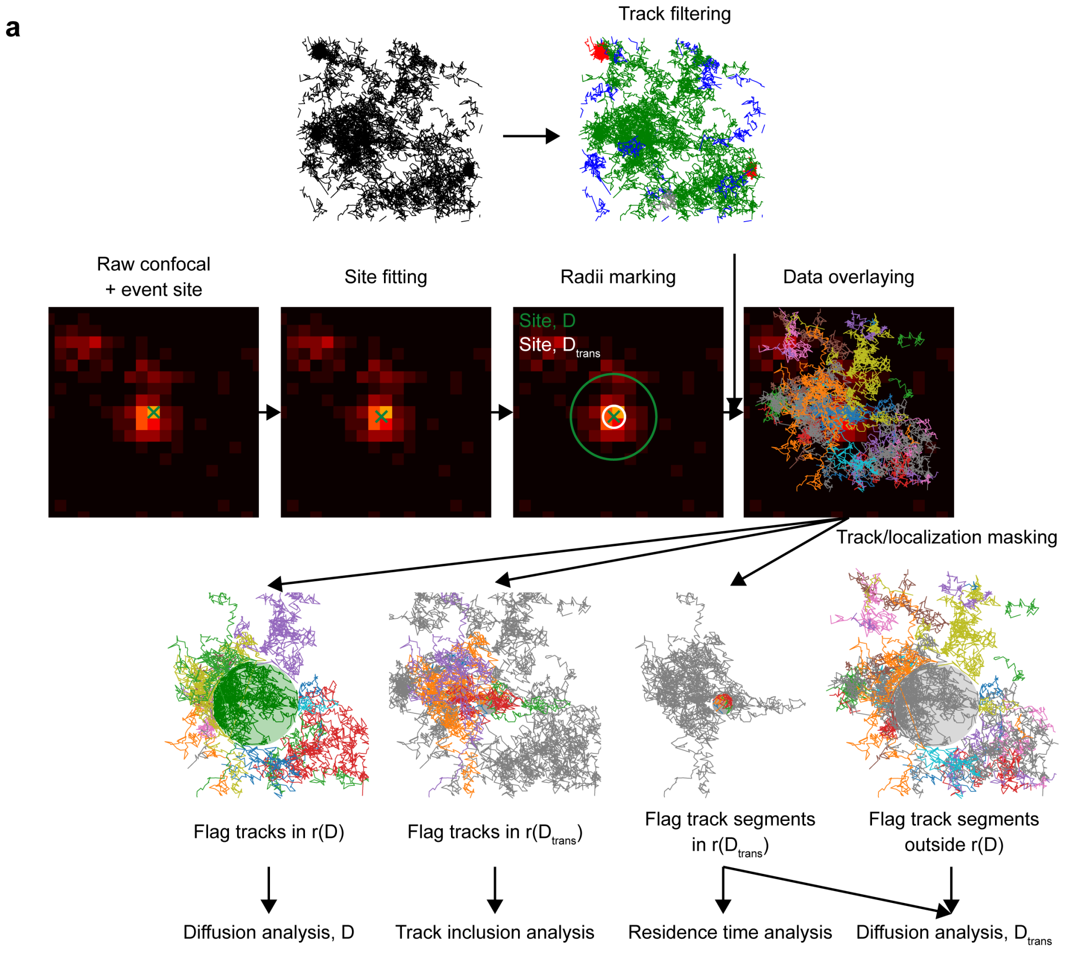

Supplementary Figure 8. Caveolin site data analysis sketch. a. Post-acquisition data analysis pipeline of Caveolin1 and random site MINFLUX tracking, showing steps of track filtering, site fitting, site radii marking, data overlaying, track and localization flagging, and parameter extraction for the analysis present in the work.

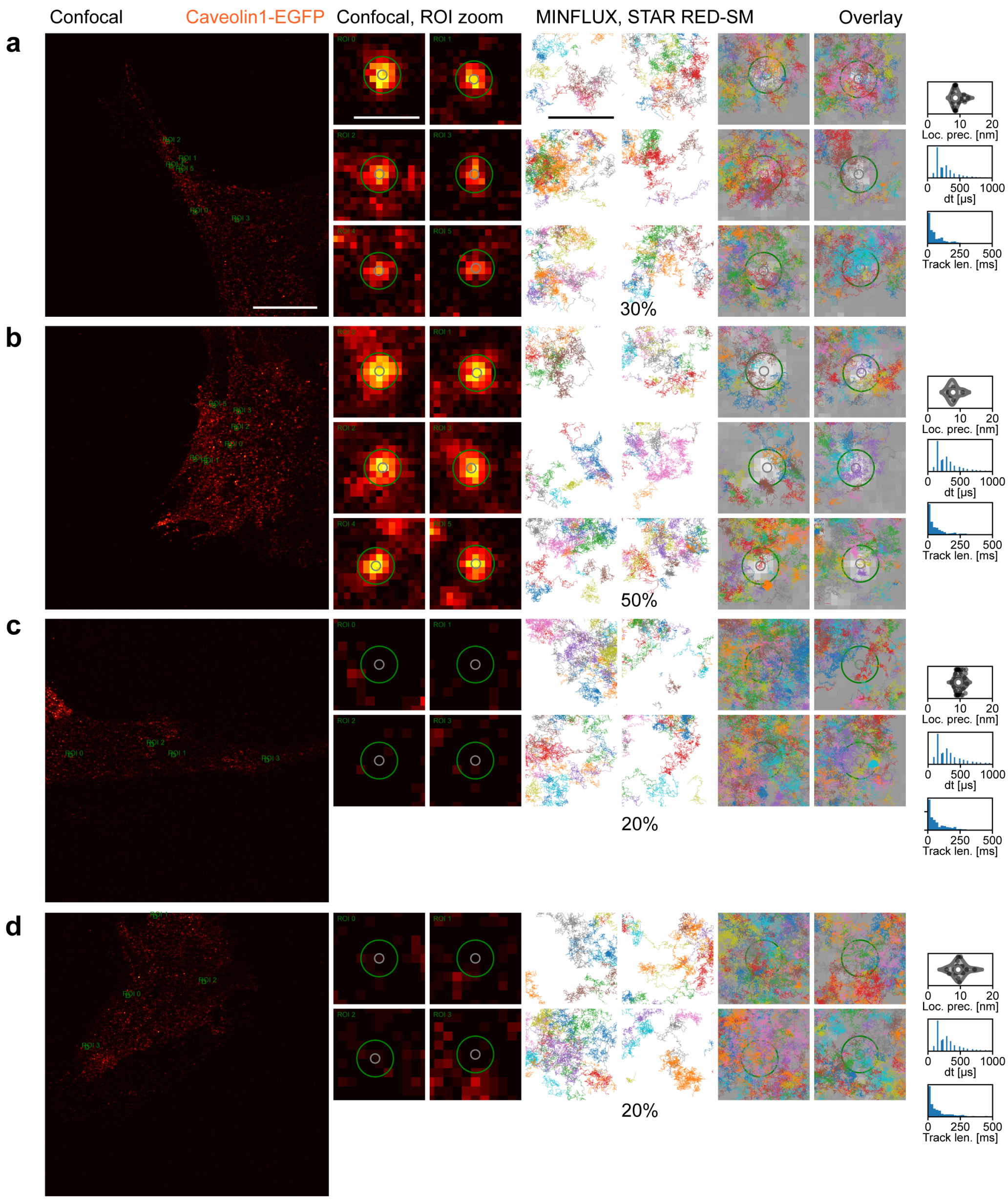

Supplementary Figure 9. etMINFLUX caveolae and random sites with SM-STAR RED – further examples. Further examples of (**a**,**b**) Caveolin1 event detection and (**c**,**d**) random site detection, and MINFLUX tracking of a lipid analogue (SM-STAR RED) in small ROIs around detected event or random sites. MINFLUX metadata populations for the shown datasets, with localization precision and temporal length per track, and time between individual localizations (far right). Scale bars: 5 µm (confocal overviews), 250 nm (confocal zooms; MINFLUX tracks). MINFLUX subsampling: in **a–d**, MINFLUX tracks plots show 30%, 50%, or 20% of all tracks from MINFLUX acquisition, as indicated in the plots.

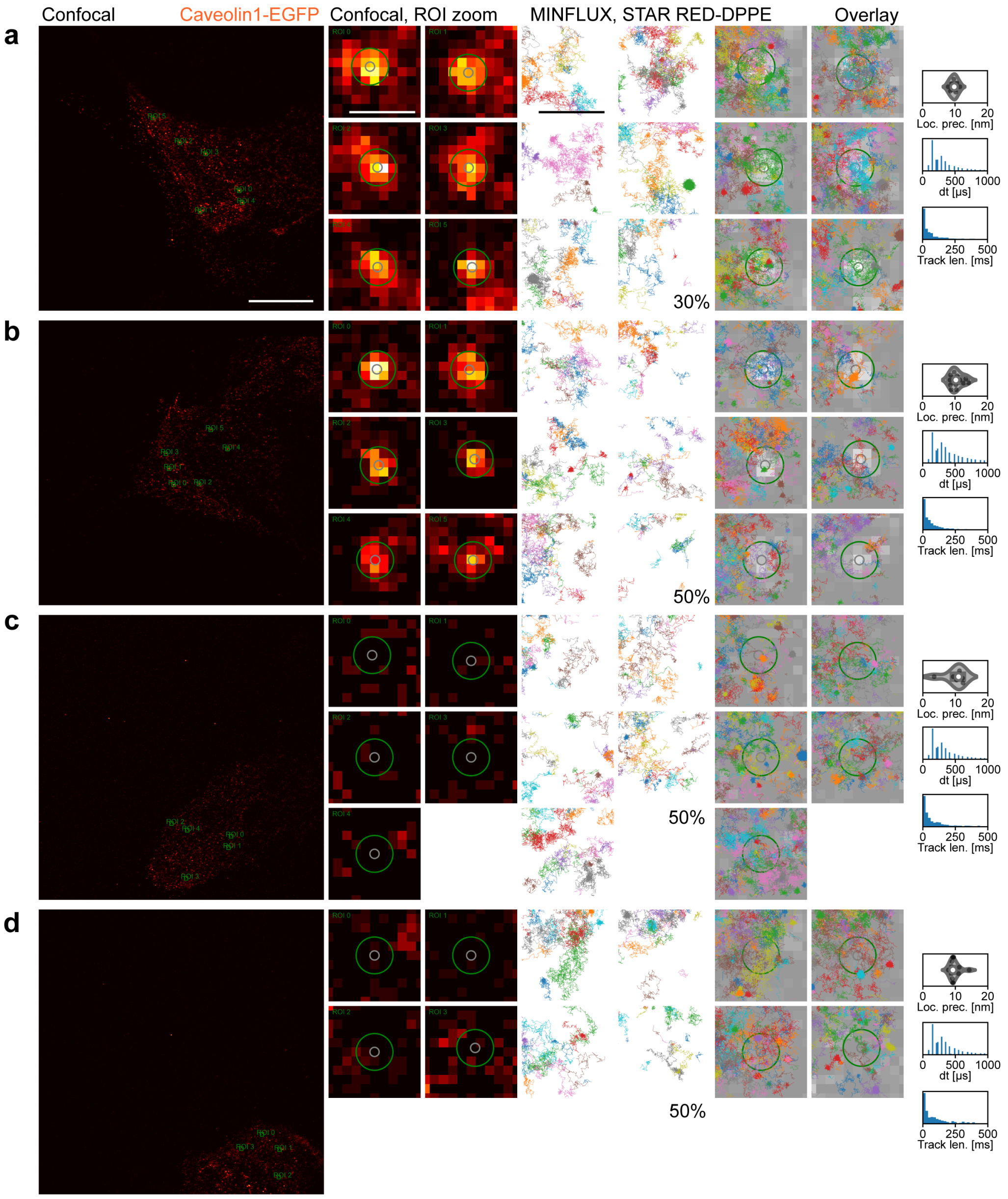

Supplementary Figure 10. etMINFLUX caveolae and random sites with DPPE-STAR RED – further examples. Further examples of (**a**,**b**) Caveolin1 event detection and (**b**,**d**) random site detection, and MINFLUX tracking of a lipid analogue (DPPE-STAR RED) in small ROIs around detected event or random sites. MINFLUX metadata populations for the shown datasets, with localization precision and temporal length per track, and time between individual localizations (far right). Scale bars: 5 μm (confocal overviews), 250 nm (confocal zooms; MINFLUX tracks). MINFLUX subsampling: in **a**–**d**, MINFLUX tracks plots show 30% or 50% of all tracks from MINFLUX acquisition, as indicated in the plots.

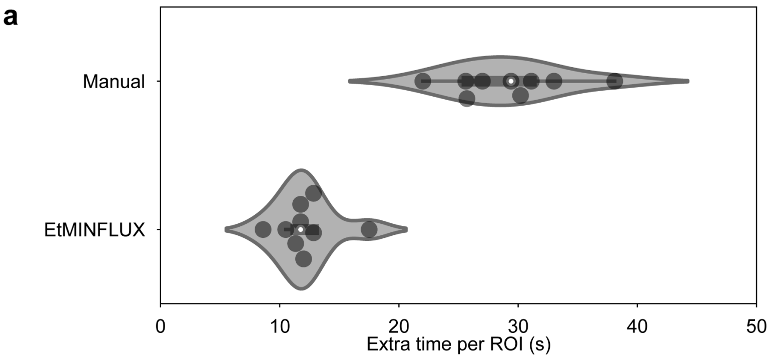

Supplementary Figure 11. Temporal data throughput for Caveolin1 experiments. a. Temporal data throughput, in terms of extra time per MINFLUX-acquired region of interest spent on overhead, in seconds, for manual vs etMINFLUX acquisitions. etMINFLUX: n=42 ROIs, n=9 cells, n=3 independent experiments. Manual: n=80 ROIs, n=9 cells, n=3 independent experiments. Violin plots: white point shows the median, box spans the IQR, and whiskers extend 1.5×IQR. Source data are provided as a Source Data file. Source data are provided as a Source Data file.

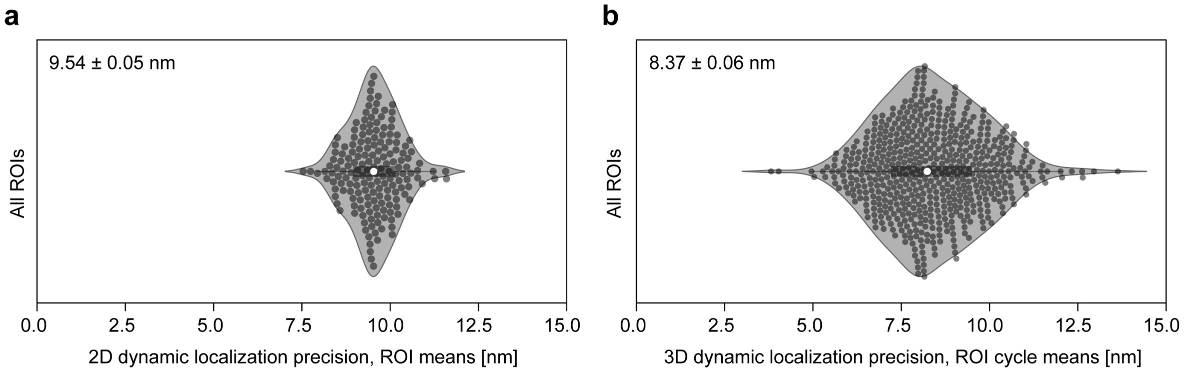

Supplementary Figure 12. MINFLUX localization precision. Dynamic localization precisions, extracted as the y-axis intercept from SD model fitting, for the various MINFLUX acquisition settings for the experiments on Caveolin1 accumulation sites and Gag accumulation sites. Each datapoint represents the mean of all SD fits in a single ROI or ROI cycle. **a.** 2D dynamic localization precision for 2D MINFLUX tracking of SM-STAR RED or DPPE-STAR RED in Caveolin1-triggered or random site experiments (all datasets combined). n=175 individual ROIs from n=8 independent experiments. **b.** 3D dynamic localization precision for 3D MINFLUX tracking of STAR RED-membrane in Gag-triggered site experiments. n=270 individual ROIs from n=10 independent experiments. Violin plots: white point shows the median, box spans the IQR, and whiskers extend 1.5×IQR. Source data are provided as a Source Data file. Source data are provided as a Source Data file.

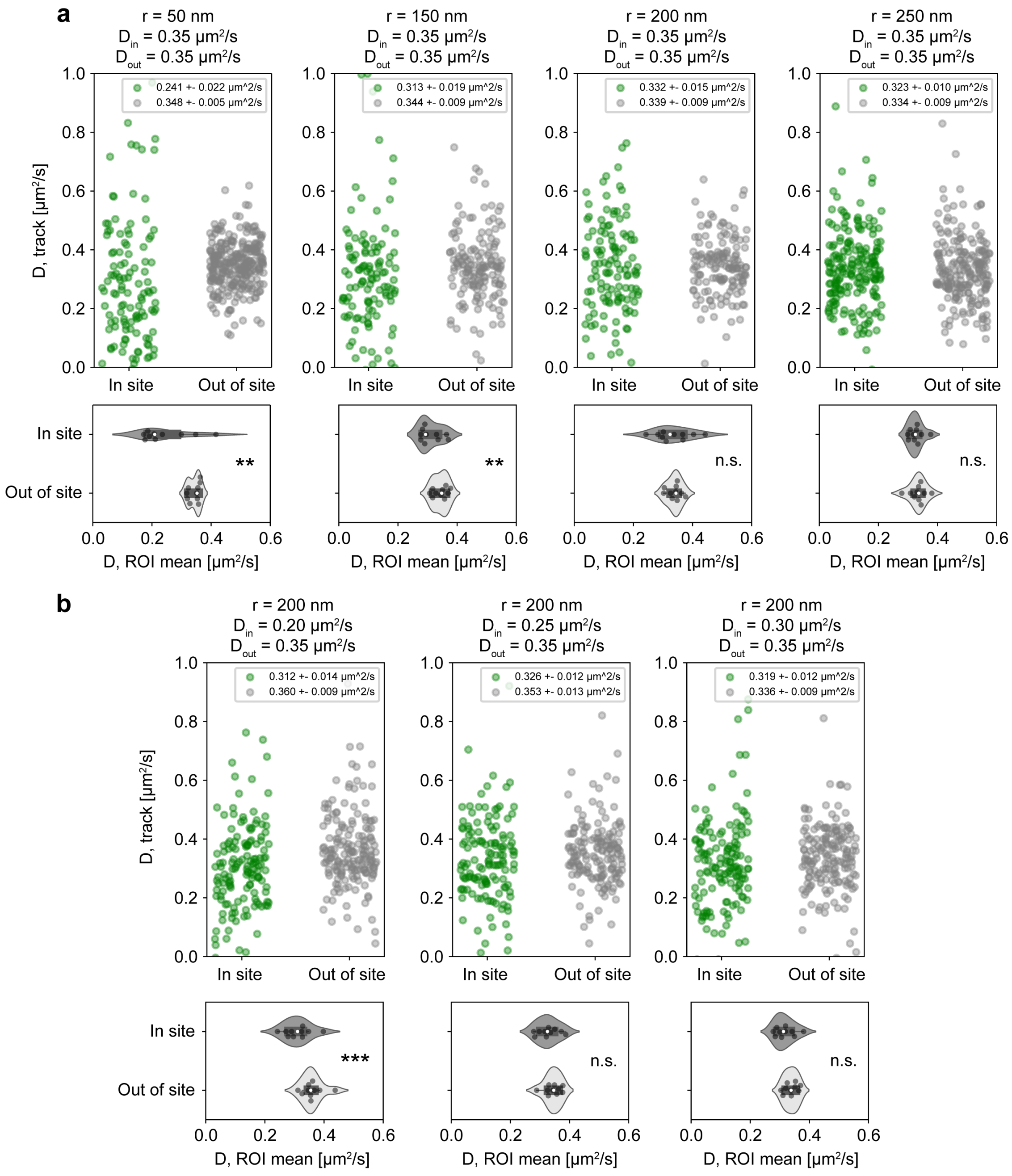

Supplementary Figure 13. Diffusion analysis on simulated data. Results from diffusion coefficient analysis performed on simulated data with various parameter combinations. **a.** Varying analysis site radius, from 50 nm to 250 nm, showing bias in diffusion coefficient estimation for smaller site radii. **b.** Varying simulated inner diffusion coefficient, from 0.20 to 0.35 µm^2/s, showing sensitivity to detecting diffusion coefficient variations for smaller values. Statistical tests: related 2-sample Student's t-test for identical expected values. p-values: (**a**) r = 50: p = 0.0060; r = 150: p = 0.0084; r = 200: p = 0.86; r = 250: p = 0.41, (**b**) D_{in}=0.20: p = 0.00086; D_{in} = 0.25: p = 0.35; D_{in} = 0.30: p = 0.096. For each condition either 10 ROIs, each with 100 tracks, each with a mean of 200 localizations, or 20 ROIs, each with 250 tracks, each with a mean of 200 localizations, were simulated. Violin plots: white point shows the median, box spans the IQR, and whiskers extend 1.5×IQR. Source data are provided as a Source Data file.

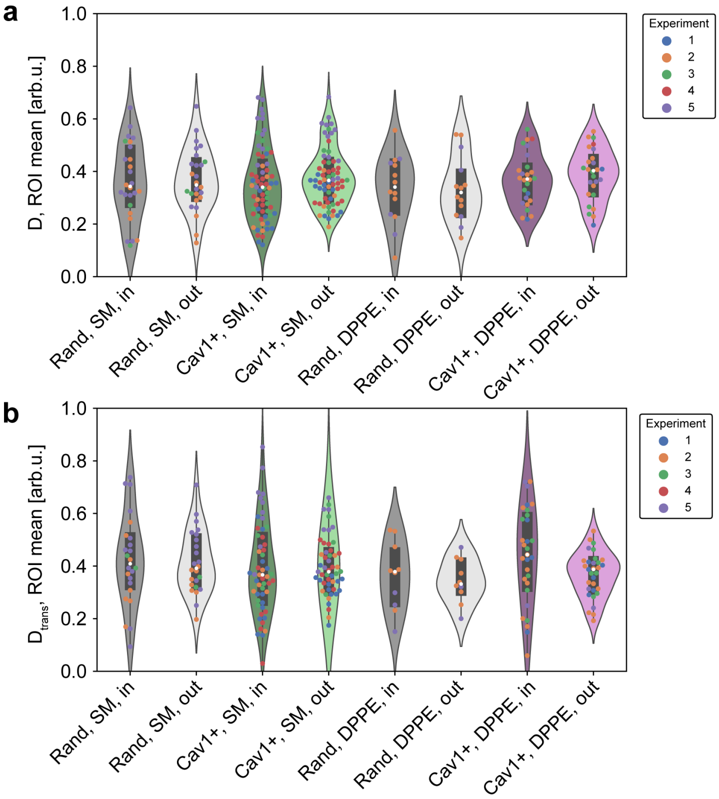

Supplementary Figure 14. Diffusion analysis results from Caveolin1 accumulation sites. Results from diffusion coefficient and transient diffusion coefficient analysis, showing the extracted diffusion coefficients inside and outside the site. Each datapoint represents the mean from all tracks in a ROI, and different experiments are color-coded. Groupings, from left to right, are inside and outside randomized sites for SM tracking, inside and outside Caveolin1-triggered sites for SM tracking, inside and outside randomized sites for DPPE tracking, and inside and outside Caveolin1-triggered sites for DPPE tracking. **a.** Diffusion coefficient analysis. **b.** Transient diffusion coefficient analysis. Each datapoint represents the mean of one caveolae site. Random SM (gray): n=306 tracks in, n=26 ROIs, n=3 independent experiments; Cav1+ SM (green): n=1157 tracks in, n=69 ROIs, n=5 independent experiments; Random DPPE (gray): n=93 tracks in, n=17 ROIs, n=3 independent experiments; Cav1+ DPPE (magenta): n=264 tracks in, n=26 ROIs, n=5 independent experiments. Violin plots: white point shows the median, box spans the IQR, and whiskers extend 1.5×IQR. Source data are provided as a Source Data file. Source data are provided as a Source Data file.

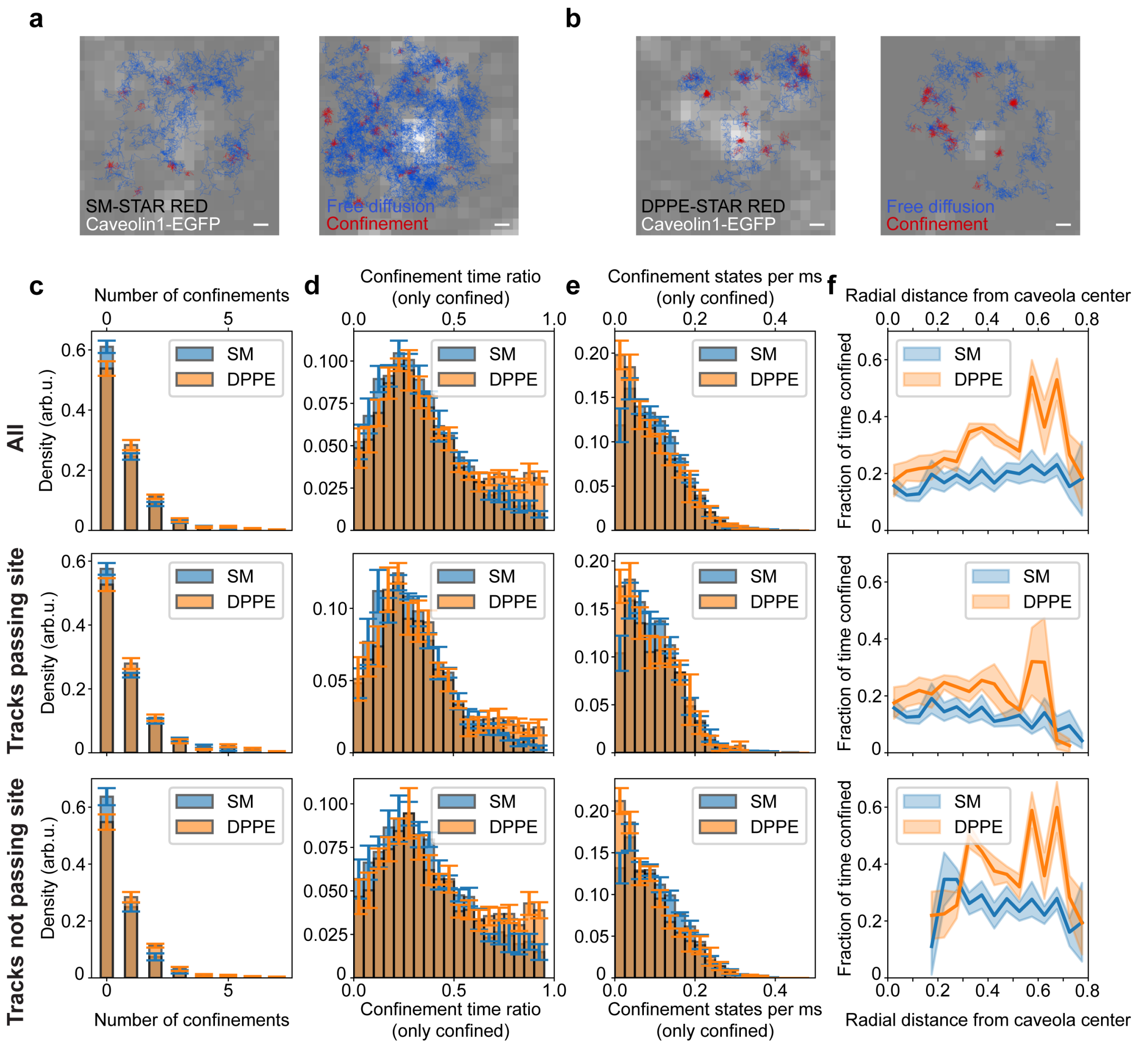

Supplementary Figure 15. Packing coefficient analysis of SM and DPPE diffusion. a, b. Example packing coefficient analysis results, for SM (a) and DPPE (b), showing free diffusion (blue) and confined states (red) as classified according to the packing coefficient. **c.** Histogram of the population of number of confinements per track, for SM (blue) and DPPE (orange), as the mean ± SEM for n=2371 tracks from n=5 individual experiments. **d.** Histogram of the population of the confinement time ratio, for only confined tracks, for SM (blue) and DPPE (orange), as the mean ± SEM for n=2371 tracks from n=5 individual experiments. **e.** Histogram of the population of number of confinement states per ms, for SM (blue) and DPPE (orange), as the mean ± SEM for n=2371 tracks from n=5 individual experiments. **f.** Mean radial profile of the fraction of time spent confined inside a region, for the radial distance from the event site center, for SM (blue) and DPPE (orange). The standard error of the mean is shown as a shaded area. n=2371 tracks from n=5 individual experiments **c-f.** The first row of plots shows the distributions for all tracks, the second row shows the distributions for only tracks that pass through the event site (as defined with a radius of 200 nm), and the third row shows the distributions for only tracks that do not pass through the event site. Scale bars: 100 nm (**a**, **b**). Source data are provided as a Source Data file.

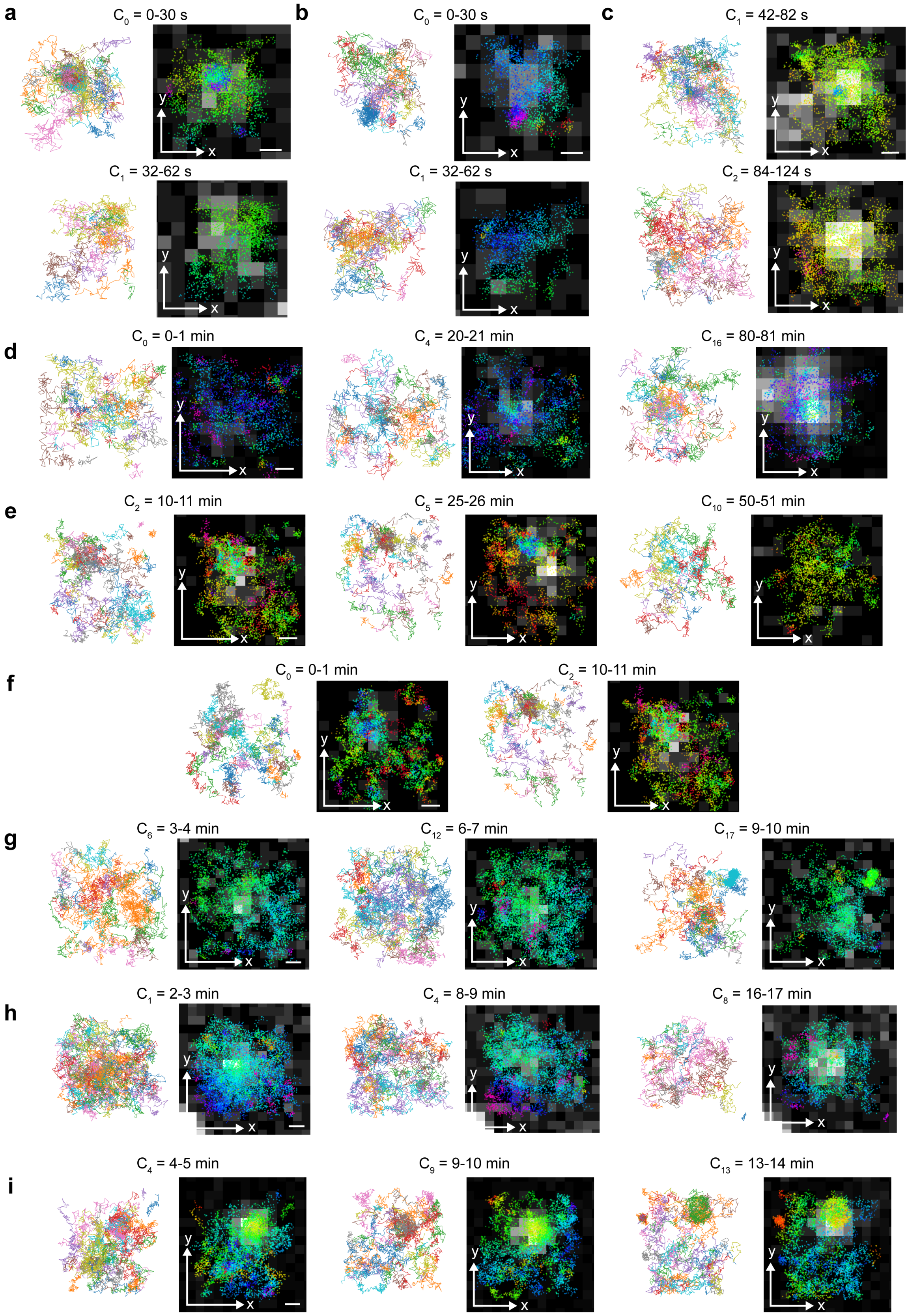

Supplementary Figure 16. Full MINFLUX datasets of previous plots with subsampled MINFLUX plots. a. Plots from Figure 4f with full MINFLUX datasets. **b.** Plots from Extended Data Figure 6b with full MINFLUX datasets. **c.** Plots from Extended Data Figure 6d with full MINFLUX datasets. **d.** Plots

from Figure 5g with full MINFLUX datasets. **e.** Plots from Figure 5h with full MINFLUX datasets. **f.** Plots from Extended Data Figure 8a with full MINFLUX datasets. **g.** Plots from Extended Data Figure 8b with full MINFLUX datasets. **h.** Plots from Extended Data Figure 8c with full MINFLUX datasets. **i.** Plots from Extended Data Figure 8d with full MINFLUX datasets.

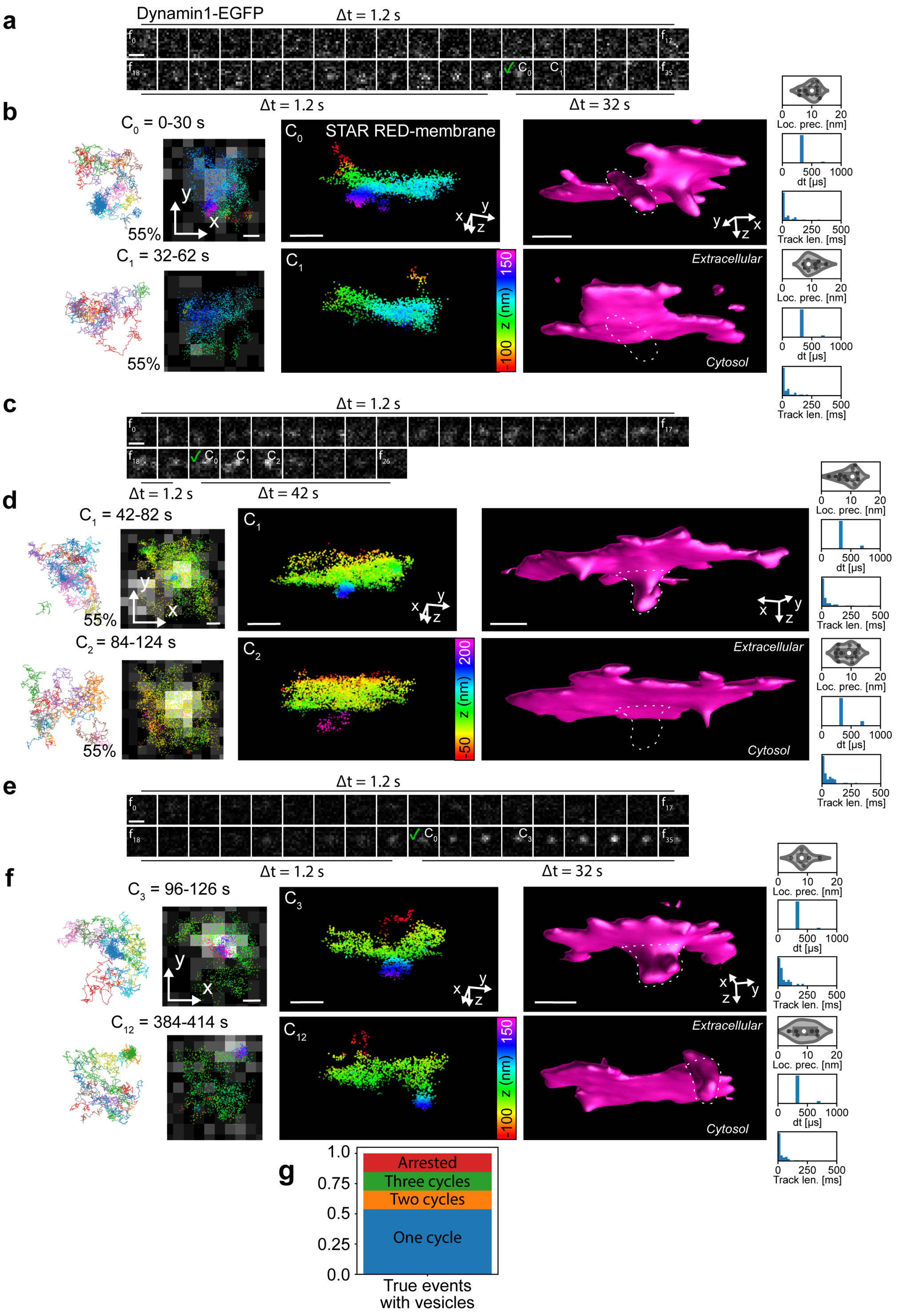

Supplementary Figure 17. etMINFLUX endocytosis sites – further examples. Further examples of etMINFLUX experiments of Dynamin1 accumulation event detection and MINFLUX tracking of a membrane marker in small ROIs around detected event sites, with topological maps of the event site. **a,**

c, e. Confocal timelapse data of event detection and during ROI follow. **b.** MINFLUX data showing endocytic vesicle present in one cycle but not in the next one. **d.** MINFLUX data showing endocytic vesicle present in two cycles, cycle 0 (not shown) and 1, but not in cycle 2. **f.** MINFLUX data showing arrested endocytic vesicle present in 12 cycles. MINFLUX metadata populations for the shown cycles, with localization precision and temporal length per track, and time between individual localizations (fifth column, **b, d, f**). Scale bars: 500 nm (**a, c, e**), 100 nm (**b, d, f**). MINFLUX subsampling: in **b** and **d**, MINFLUX tracks shows 55% of all tracks from MINFLUX acquisition, as indicated in the plots. Source data are provided as a Source Data file.

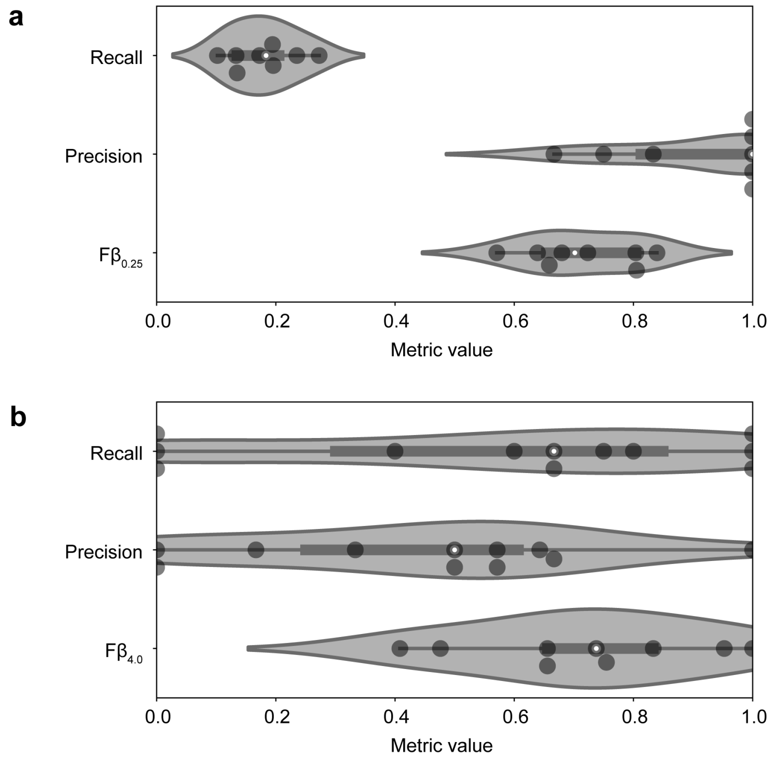

Supplementary Figure 18. Pipeline performance evaluation. a, b. Performance of dyn_signalrise (**a**) and gag_signalrise (**b**) pipelines upon a timelapse-specific Bayesian parameter optimization routine, in terms of recall ($\frac{TP}{P}$), precision ($\frac{TP}{TP+FP}$), and $F\beta$ ($(1+\beta^2)\frac{(precision \times recall)}{(\beta^2 \times precision)+recall}$). dynamin_signalrise: n=8 field-of-views from n=3 independent experiments. gag_signalrise: n=12 field-of-views from n=3 independent experiments. Violin plots: white point shows the median, box spans the IQR, and whiskers extend 1.5×IQR. Source data are provided as a Source Data file. Source data are provided as a Source Data file.

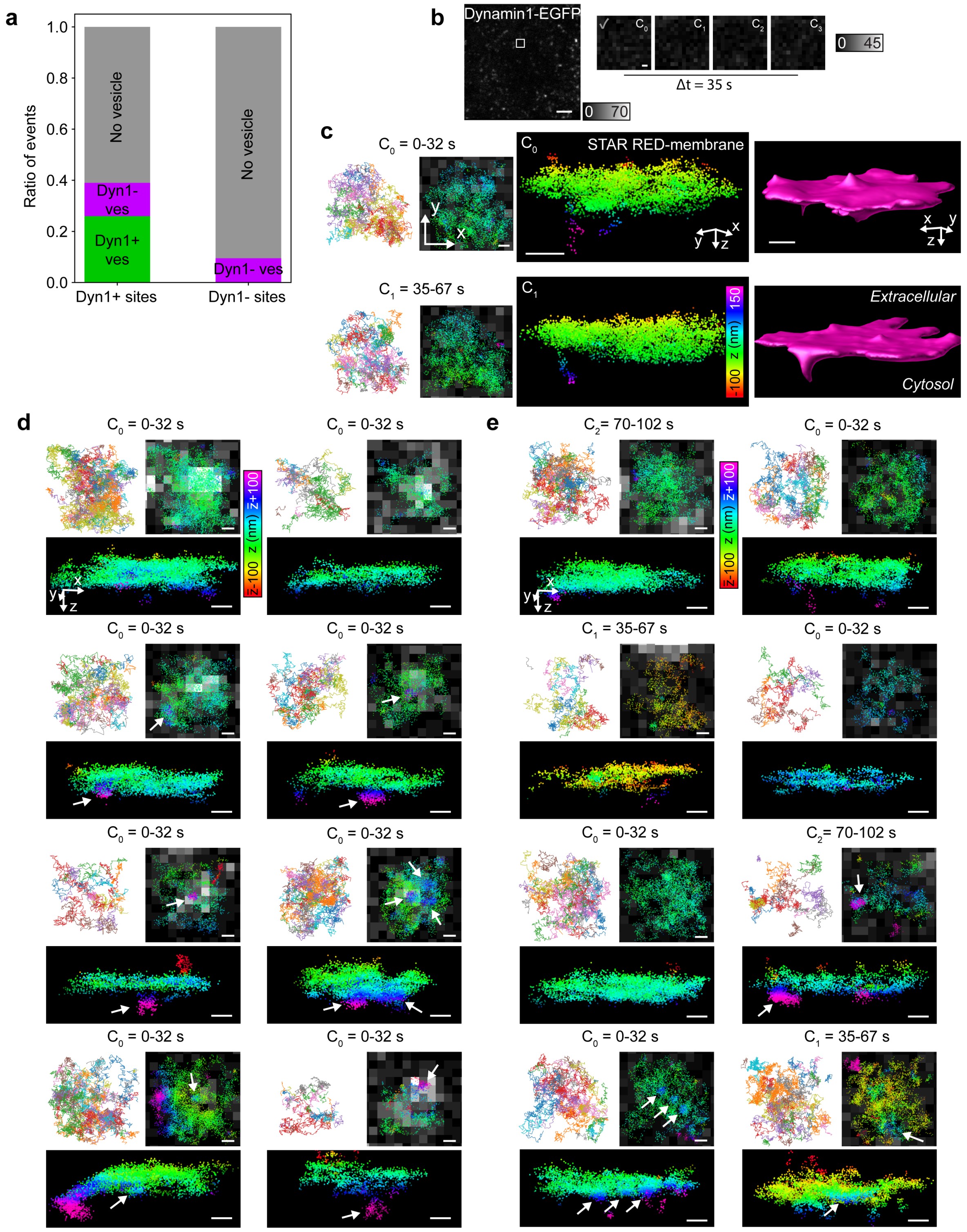

Supplementary Figure 19. Live imaging of detected Dynamin1- and comparison to Dynamin1+ sites. a. Statistics on ratio of detected events with either a Dynamin1-overlapping vesicle (green) or a non-Dynamin1-overlapping vesicle (magenta) visible in the 3D MINFLUX tracking data, compared to all true Dynamin1+ or Dynamin1- event detections. **b.** Example of Dynamin1- event detection, with large confocal image showing Dynamin1-EGFP (left) and zoom-ins to the event area in a time lapse collage (right). Frame triggering event is marked with a check mark. C_i indicates frame before which MINFLUX cycle i was recorded. **c.** Exemplary MINFLUX data of the event shown in **b**, showing two recording cycles (cycle 0 and 1) of the same event. Each cycle shows tracks of STAR RED-membrane

(first column), MINFLUX localizations overlaid on confocal zoom-in of event area (top row, second column), and 3D localization point cloud with color-coded z coordinate (third column). MINFLUX data of two timepoints are shown as surface fittings to the 3D localization point cloud (fourth column). **d.** Matrix of 2D track plots, 2D scatter plots of projected MINFLUX localizations overlapping respective confocal image, and 3D scatter plots for detected Dynamin1+ events. **e.** Matrix of 2D track plots, 2D scatter plots of projected MINFLUX localizations overlapping respective confocal image, and 3D scatter plots for detected Dynamin1- events. Scale bars: 2 µm (**b** large), 100 nm (**b** zooms, **c**, **d**, **e**). Source data are provided as a Source Data file.

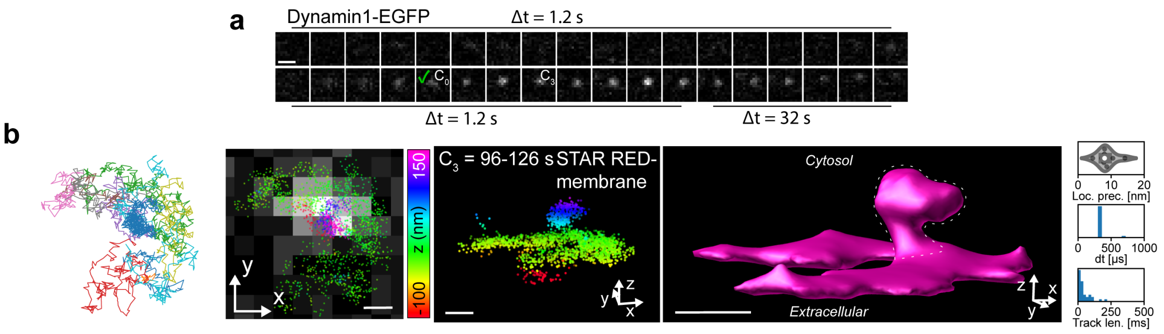

Supplementary Figure 20. etMINFLUX endocytosis site with long endosome neck. A further example of a Dynamin1 accumulation event detection and MINFLUX tracking of a membrane marker in a small ROI around the detected event site. **a.** Confocal timelapse data of event detection and during ROI following. **b.** MINFLUX tracking data from the event, represented as tracks, localization clouds in 2D and 3D, as well as a topological map of the membrane surface. MINFLUX metadata populations for the shown cycle, with localization precision and temporal length per track, and time between individual localizations (fifth column). Scale bars: 500 nm (**a**), 100 nm (**b**).

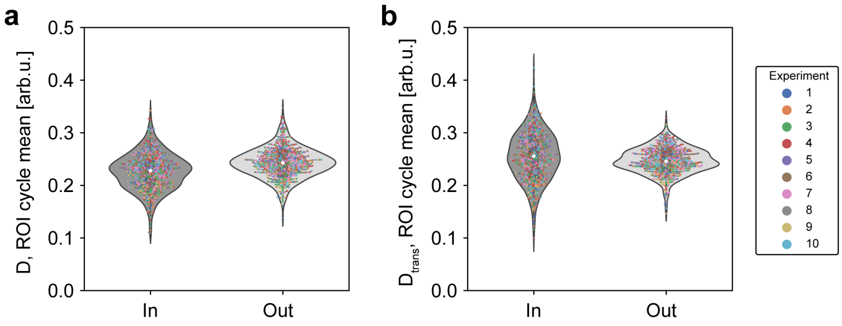

Supplementary Figure 21. Diffusion coefficient and transient diffusion coefficient analysis results from Gag accumulation sites. Results from diffusion coefficient and transient diffusion coefficient analysis, showing the extracted diffusion coefficients inside and outside the site. Each datapoint represents the mean from all tracks in a ROI cycle, and different experiments are color-coded. **a.** Diffusion coefficient analysis. n=643 ROI cycles from n=54 ROIs from n=10 independent experiments. **b.** Transient diffusion coefficient analysis. n=643 ROI cycles from n=54 ROIs from n=10 independent experiments. Violin plots: white point shows the median, box spans the IQR, and whiskers extend 1.5×IQR. Source data are provided as a Source Data file. Source data are provided as a Source Data file.

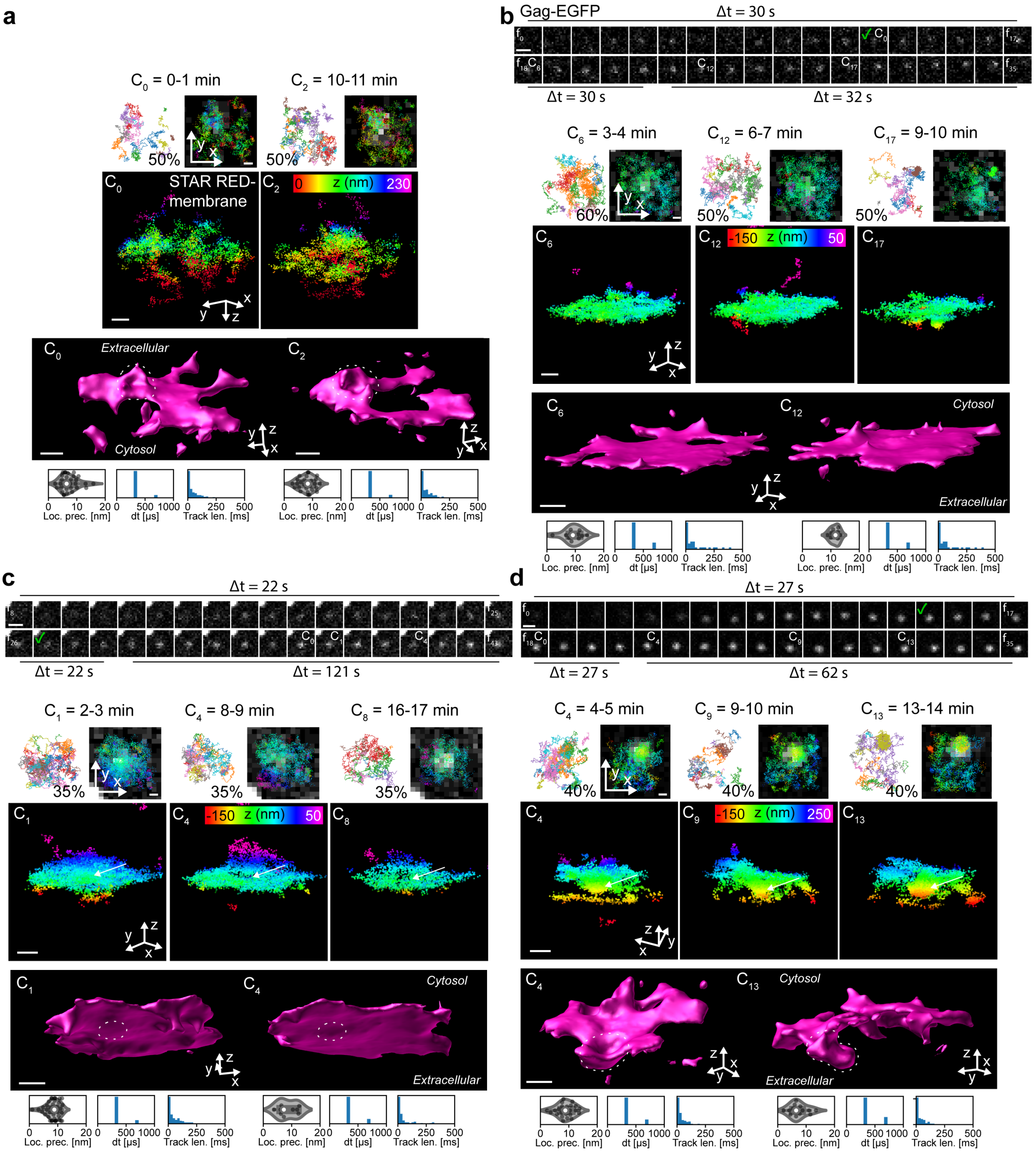

Supplementary Figure 22. etMINFLUX virus budding sites – further examples. Further examples of Gag accumulation event detection and MINFLUX tracking of a membrane marker in small ROIs around detected event sites, with topological maps of the event site. **a.** Additional time points of the event shown in Figure 5f,h. **b.** An event with no sign of budding. **c.** An event with moderate static bulging. **d.** An event with a developing budding site. MINFLUX metadata populations for the two cycles shown as 3D surfaces, with localization precision and temporal length per track, and time between individual localizations (bottom in all panels). Scale bars: 500 nm (confocal time lapse in **b–d**), 100 nm (MINFLUX localizations-confocal overlay, 3D MINFLUX localizations, and 3D MINFLUX surface in **a–d**). MINFLUX subsampling: in **a–d**, MINFLUX tracks plots show 35%, 40%, 50% or 60% of all from MINFLUX acquisition, as indicated in the plots.

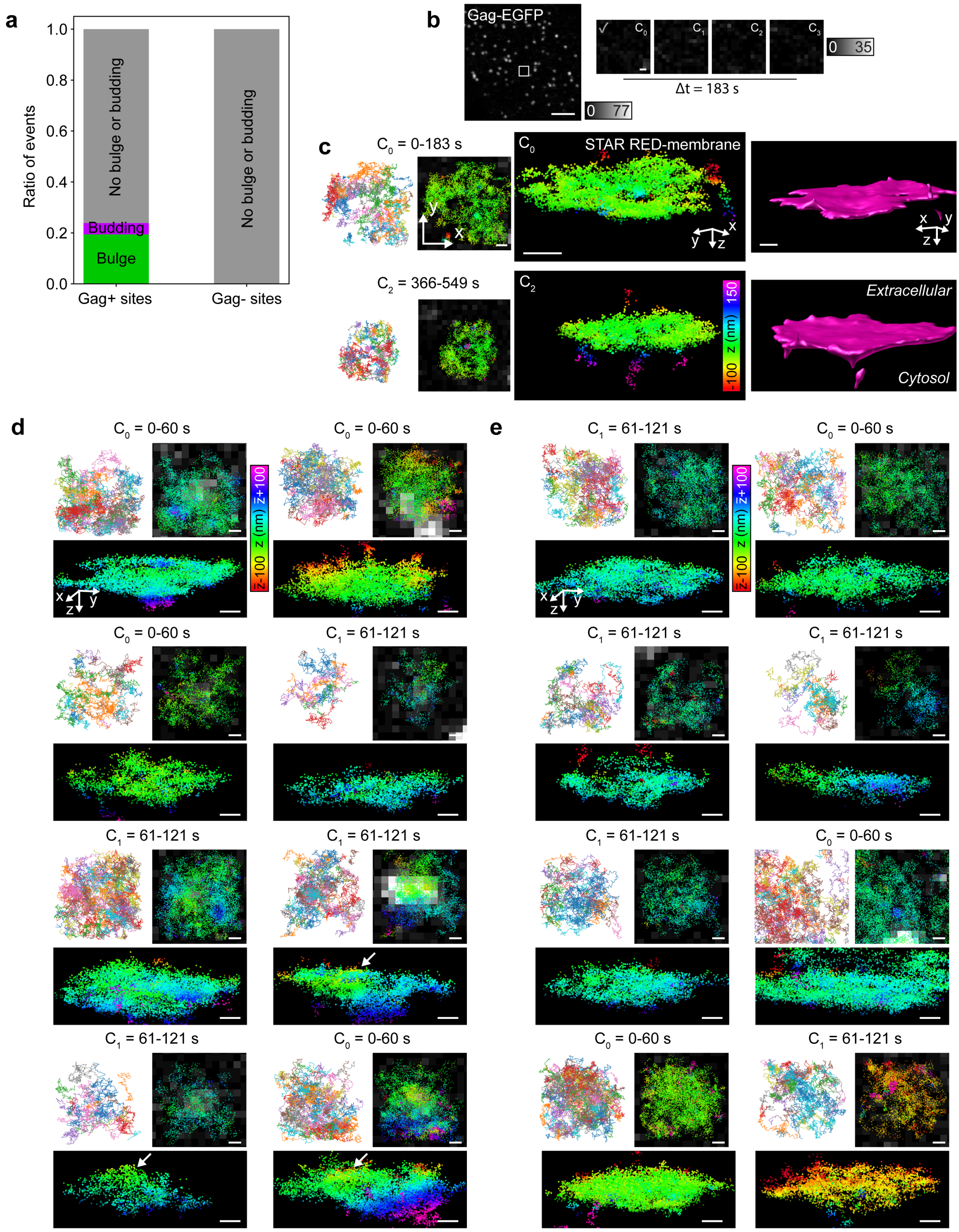

Supplementary Figure 23. Live imaging of detected Gag- and comparison to Gag+ sites. a. Statistics on number of detected events with either a static bulging membrane (green) or a dynamically budding membrane (magenta) visible in the 3D MINFLUX tracking data, compared to all true Gag+ or Gag- event detections. **b.** Example of Gag- event detection, with large confocal image showing Gag-EGFP (left) and zoom-ins to the event area in a time lapse collage (right). Frame triggering event is marked with a check mark. C_i indicates frame before which MINFLUX cycle i was recorded. **c.** Exemplary MINFLUX data of the event shown in **b**, showing two recording cycles (cycle 0 and 2) of the same event. Each cycle shows tracks of STAR RED-membrane (first column), MINFLUX

localizations overlaid on confocal zoom-in of event area (top row, second column), and 3D localization point cloud with color-coded z coordinate (third column). MINFLUX data of two timepoints are shown as surface fittings to the 3D localization point cloud (fourth column). **d.** Matrix of 2D track plots, 2D scatter plots of projected MINFLUX localizations overlapping respective confocal image, and 3D scatter plots for detected Gag+ events. **e.** Matrix of 2D track plots, 2D scatter plots of projected MINFLUX localizations overlapping respective confocal image, and 3D scatter plots for detected Gag- events. Scale bars: 2 µm (**b** large), 100 nm (**b** zooms, **c**, **d**, **e**). Source data are provided as a Source Data file.

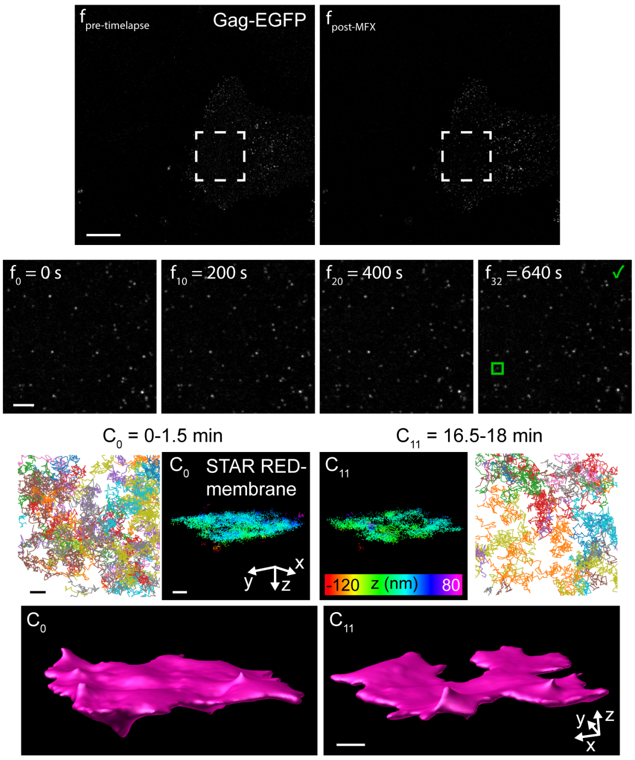

Supplementary Figure 24. Manual Gag+ event site recording. Confocal and MINFLUX data from manual Gag+ event site recording, recorded as a MINFLUX timelapse of STAR RED-membrane after manual detection of a Gag accumulation site. Complete FOV confocal images before event detection time lapse and after MINFLUX timelapse (top row), confocal images from the event detection time lapse (second row), MINFLUX data of two timepoints shown as 2D tracks of tracks and 3D localization point clouds (third row), and MINFLUX data of two timepoints shown as surface fittings to the 3D localization point clouds (bottom row). Scale bars: 10 µm (overviews), 2 µm (confocal images), 100 nm (MINFLUX tracks, 3D MINFLUX localizations, and 3D MINFLUX surfaces).

Supplementary Tables

Supplementary Table 1. etMINFLUX analysis pipeline parameter value ranges.

**peak_detection**													
**maxfilter_kersize**	**min_dist**	**th_abs**	**sm_rad**	**border_lim**	**init_sm**	**roi_border**	**roi_th_factor**						
5.0	8.0–30.0	2.0–10.0	1.0	15	True	5.0	6.0						
**dyn_signalrise**													
**min_dist**	**num_peaks**	**th_abs_lo**	**th_abs_hi**	**border_lim**	**mem_frames**	**tr_s_dist**	**frames_app**	**th_incrat**	**th_incrat_max**	**th_move_dist**			
1.0	200–1000	0.4–1.3	10.0–15.0	15	10	6–8	4–7	1.17–1.3	5	1.4–1.5			
**gag_signalrise**													
**min_dist_app**	**num_peaks**	**th_abs_lo**	**th_abs_hi**	**finalint_lo**	**finalint_hi**	**border_lim**	**mem_frames**	**tr_s_dist**	**frames_app**	**th_incrat**	**th_incrat_max**	**incslope**	**th_move_dist**
1.0–5.0	100–500	1.3–5.0	10–300	0.75	5.0–50.0	10	5–6	10	3–6	1.1–1.3	5.0–15.0	0.05–0.1	1.3–3

Supplementary Table 2. Confocal and MINFLUX acquisition parameters in the various experiments.

	Confocal						MINFLUX			
**Experiment**	**Exc. power, 488 nm, sample [µW]**	**ROI size [µm²]**	**Pixel size [nm]**	**Pixel dwell time [µs]**	**Frame time [s]**	**Frame period [s]**	**Exc. power, it. 1, 640 nm, sample [µW]**	**ROI size [µm²]**	**Cycle time [s]**	**Cycle period [s]**
Caveolin1	1–10	15×15 or 20×20 or 80×80	70 or 100	2	0.3 or 0.4 or 1.9 or 3.2	-	20–60	1–2×1–2	52–90	-
Dynamin1	0.6–3.0	10×10 or 15×15	70	20	0.6 or 1.2	0.6–1.2	39–40.5	0.5–0.8×0.5–0.8	20–40	21–42
Gag	0.7–2.7	10–25×10–25	60	10	0.5–2.1	20–30	39.0–39.9	0.7–0.8×0.7–0.8	30–60	31–298

Supplementary References

1. Max Planck Institute for Biophysical Chemistry & Abberior Instruments GmbH. specpy: Python Interface for Imspector. (2016).
2. mouse: Hook and simulate mouse events on Windows and Linux.
3. pynput: Monitor and control user input devices.
4. Alvelid, J., Damenti, M., Sgattoni, C. & Testa, I. Event-triggered STED imaging. *Nat Methods* **19**, 1268–1275 (2022).
5. Harris, C. R. *et al.* Array programming with NumPy. *Nature* **585**, 357–362 (2020).
6. Virtanen, P. *et al.* SciPy 1.0: fundamental algorithms for scientific computing in Python. *Nat Methods* **17**, 261–272 (2020).
7. McKinney, W. Data Structures for Statistical Computing in Python. in 56–61 (Austin, Texas, 2010). doi:10.25080/Majora-92bf1922-00a.
8. Van Der Walt, S. *et al.* scikit-image: image processing in Python. *PeerJ* **2**, e453 (2014).
9. Bradski, G. The OpenCV Library. *Dr. Dobb's Journal of Software Tools* **120**, 122–125 (2000).
10. The pandas development team. pandas-dev/pandas: Pandas. (2020).
11. Allan, D. B., Caswell, T., Keim, N. C., van der Wel, C. M. & Verweij, R. W. soft-matter/trackpy: Trackpy. (2021).
12. Michalet, X. & Berglund, A. J. Optimal diffusion coefficient estimation in single-particle tracking. *Phys Rev E Stat Nonlin Soft Matter Phys* **85**, 061916 (2012).
13. Balzarotti, F. *et al.* Nanometer resolution imaging and tracking of fluorescent molecules with minimal photon fluxes. *Science* **355**, 606–612 (2017).
14. Ester, M., Kriegel, H.-P., Sander, J. & Xu, X. A density-based algorithm for discovering clusters in large spatial databases with noise. *Proceedings of the Second International Conference on Knowledge Discovery and Data Mining (KDD-96)* 226–231 (1996) doi:10.1.1.121.9220.
15. Pedregosa, F. *et al.* Scikit-learn: Machine Learning in Python. *Journal of Machine Learning Research* **12**, 2825–2830 (2011).
