# Supplementary material for: Smart event-triggered MINFLUX microscopy to catch and follow rare events
Source: Nat Commun. 2026 May 21;17:4558. doi: 10.1038/s41467-026-73176-z (PMC13195108; doi:10.1038/s41467-026-73176-z)
Supplement: Supplementary file 2 — Reporting Summary [file 41467_2026_73176_MOESM2_ESM.pdf]

nature portfolio

Corresponding author(s): Jonatan Alvelid, Christian Eggeling

Last updated by author(s): Apr 23, 2026

Reporting Summary

Nature Portfolio wishes to improve the reproducibility of the work that we publish. This form provides structure for consistency and transparency in reporting. For further information on Nature Portfolio policies, see our Editorial Policies and the Editorial Policy Checklist.

Statistics

For all statistical analyses, confirm that the following items are present in the figure legend, table legend, main text, or Methods section.

n/a	Confirmed	
☐	☒	The exact sample size (*n*) for each experimental group/condition, given as a discrete number and unit of measurement
☐	☒	A statement on whether measurements were taken from distinct samples or whether the same sample was measured repeatedly
☐	☒	The statistical test(s) used AND whether they are one- or two-sided<br>*Only common tests should be described solely by name; describe more complex techniques in the Methods section.*
☒	☐	A description of all covariates tested
☒	☐	A description of any assumptions or corrections, such as tests of normality and adjustment for multiple comparisons
☐	☒	A full description of the statistical parameters including central tendency (e.g. means) or other basic estimates (e.g. regression coefficient) AND variation (e.g. standard deviation) or associated estimates of uncertainty (e.g. confidence intervals)
☐	☒	For null hypothesis testing, the test statistic (e.g. *F*, *t*, *r*) with confidence intervals, effect sizes, degrees of freedom and *P* value noted<br>*Give P values as exact values whenever suitable.*
☒	☐	For Bayesian analysis, information on the choice of priors and Markov chain Monte Carlo settings
☒	☐	For hierarchical and complex designs, identification of the appropriate level for tests and full reporting of outcomes
☒	☐	Estimates of effect sizes (e.g. Cohen's *d*, Pearson's *r*), indicating how they were calculated

Our web collection on statistics for biologists contains articles on many of the points above.

Software and code

Policy information about availability of computer code

Data collection	The MINFLUX measurements were performed on a commercial abberior MINFLUX system using the abberior Imspector microscope control software, version 16.3.15635-m2205, and the etMINFLUX software, version 1.0.0, which was developed in the scope of this study, to control Imspector. The etMINFLUX control of Imspector took place using the Imspector API module specpy (v1.2.3), and help using the Python modules mouse (v0.7.1) and pynput (v1.7.7). A newer version of the software etMINFLUX is released with this article (v1.3.0) and has been tested using Imspector version 16.3.21315-m2410.
Data analysis	Data analysis was performed with custom-written scripts in Python (v3.10.4 and v3.10.11), with packages numpy (v1.24.3), scipy (v1.10.1), scikit-image (v0.23.1), scikit-learn (v1.2.2), matplotlib (v3.7.1), opencv (v4.6.0), pandas (v1.5.3), seaborn (v0.12.2), tifffile (v2023.4.12), specpy (v1.2.3), trackpy (v0.6.2). The scripts are available at https://www.github.com/jonatanalvelid/etMINFLUX-analysis-public. Data visualization has also been performed with the custom-written napari plugin napari-etminflux-data-viewer (v.1.0.2): https://github.com/jonatanalvelid/napari-etminflux-data-viewer.

For manuscripts utilizing custom algorithms or software that are central to the research but not yet described in published literature, software must be made available to editors and reviewers. We strongly encourage code deposition in a community repository (e.g. GitHub). See the Nature Portfolio guidelines for submitting code & software for further information.

Data

Policy information about availability of data

All manuscripts must include a data availability statement. This statement should provide the following information, where applicable:

- Accession codes, unique identifiers, or web links for publicly available datasets
- A description of any restrictions on data availability
- For clinical datasets or third party data, please ensure that the statement adheres to our policy

The data that support the implementation of the method and support the findings in this study, including images, log-files, and metadata, are openly available in Zenodo at https://doi.org/10.5281/zenodo.15608839, reference number 15608839.

Research involving human participants, their data, or biological material

Policy information about studies with human participants or human data. See also policy information about sex, gender (identity/presentation), and sexual orientation and race, ethnicity and racism.

Reporting on sex and gender	*Use the terms sex (biological attribute) and gender (shaped by social and cultural circumstances) carefully in order to avoid confusing both terms. Indicate if findings apply to only one sex or gender; describe whether sex and gender were considered in study design; whether sex and/or gender was determined based on self-reporting or assigned and methods used. Provide in the source data disaggregated sex and gender data, where this information has been collected, and if consent has been obtained for sharing of individual-level data; provide overall numbers in this Reporting Summary. Please state if this information has not been collected. Report sex- and gender-based analyses where performed, justify reasons for lack of sex- and gender-based analysis.*
Reporting on race, ethnicity, or other socially relevant groupings	*Please specify the socially constructed or socially relevant categorization variable(s) used in your manuscript and explain why they were used. Please note that such variables should not be used as proxies for other socially constructed/relevant variables (for example, race or ethnicity should not be used as a proxy for socioeconomic status). Provide clear definitions of the relevant terms used, how they were provided (by the participants/respondents, the researchers, or third parties), and the method(s) used to classify people into the different categories (e.g. self-report, census or administrative data, social media data, etc.) Please provide details about how you controlled for confounding variables in your analyses.*
Population characteristics	*Describe the covariate-relevant population characteristics of the human research participants (e.g. age, genotypic information, past and current diagnosis and treatment categories). If you filled out the behavioural & social sciences study design questions and have nothing to add here, write "See above."*
Recruitment	*Describe how participants were recruited. Outline any potential self-selection bias or other biases that may be present and how these are likely to impact results.*
Ethics oversight	*Identify the organization(s) that approved the study protocol.*

Note that full information on the approval of the study protocol must also be provided in the manuscript.

Field-specific reporting

Please select the one below that is the best fit for your research. If you are not sure, read the appropriate sections before making your selection.

☒ Life sciences ☐ Behavioural & social sciences ☐ Ecological, evolutionary & environmental sciences

For a reference copy of the document with all sections, see nature.com/documents/nr-reporting-summary-flat.pdf

Life sciences study design

All studies must disclose on these points even when the disclosure is negative.

Sample size	No sample size-determining statistical testing was performed. The number of samples and experiments were determined for each experiment type so as to provide enough statistical power considering sample variability and maximized in all cases. The developed method allows a significant increase in the sample size as compared to manual recordings, as highlighted in the study. All presented results derive from at least 3 experimental replicates, except pooling for summaries of the full datasets.
Data exclusions	No data were excluded from the analysis.
Replication	All individual etMINFLUX experiments as part of this study were repeated in multiple samples, in multiple experiments, over multiple days of recording. All experiments were reproducible in different days of recording and for extended times after system alignment (up to one day). System alignment was stable over time for the full day of recording. All attempts at replication were successful.
Randomization	In experiments, individual cells were randomized from the sample wells, and sample wells were randomized from the 8-well slides. The automation code randomizes event sites inside the cell so as which avoids bias in selection of event sites. In sample preparation, no different

	conditions were prepared and as such no randomization for samples into condition groups was relevant.
Blinding	No allocation into experimental groups were performed. Blinding was not possible during data collection due to the complexity of MINFLUX experiments. Blinding was neither relevant in the experiment phase, as different conditions were not compared. In data analysis, analysis was automated to run similarly on all compared populations, as such blinding was not relevant.

Reporting for specific materials, systems and methods

We require information from authors about some types of materials, experimental systems and methods used in many studies. Here, indicate whether each material, system or method listed is relevant to your study. If you are not sure if a list item applies to your research, read the appropriate section before selecting a response.

Materials & experimental systems

n/a	Involved in the study	
☒	☐	Antibodies
☐	☒	Eukaryotic cell lines
☒	☐	Palaeontology and archaeology
☒	☐	Animals and other organisms
☒	☐	Clinical data
☒	☐	Dual use research of concern
☒	☐	Plants

Methods

n/a	Involved in the study	
☒	☐	ChIP-seq
☒	☐	Flow cytometry
☒	☐	MRI-based neuroimaging

Eukaryotic cell lines

Policy information about cell lines and Sex and Gender in Research

Cell line source(s)	HeLa, ATCC CCL-2; PtK2, ATCC CCL-56
Authentication	None of the cell lines used were authenticated.
Mycoplasma contamination	All cell lines were tested negative for mycoplasma contamination.
Commonly misidentified lines (See ICLAC register)	No commonly misidentified cell lines were used in this study.

Plants

Seed stocks	*Report on the source of all seed stocks or other plant material used. If applicable, state the seed stock centre and catalogue number. If plant specimens were collected from the field, describe the collection location, date and sampling procedures.*
Novel plant genotypes	*Describe the methods by which all novel plant genotypes were produced. This includes those generated by transgenic approaches, gene editing, chemical/radiation-based mutagenesis and hybridization. For transgenic lines, describe the transformation method, the number of independent lines analyzed and the generation upon which experiments were performed. For gene-edited lines, describe the editor used, the endogenous sequence targeted for editing, the targeting guide RNA sequence (if applicable) and how the editor was applied.*
Authentication	*Describe any authentication procedures for each seed stock used or novel genotype generated. Describe any experiments used to assess the effect of a mutation and, where applicable, how potential secondary effects (e.g. second site T-DNA insertions, mosiacism, off-target gene editing) were examined.*
