# Supplementary material for: Smart event-triggered MINFLUX microscopy to catch and follow rare events
Source: Nat Commun. 2026 May 21;17:4558. doi: 10.1038/s41467-026-73176-z (PMC13195108; doi:10.1038/s41467-026-73176-z)
Supplement: Supplementary file 3 — Description of additional supplementary files [file 41467_2026_73176_MOESM3_ESM.pdf]

DESCRIPTION OF ADDITIONAL SUPPLEMENTARY FILES

Supplementary Data 1 - Light microscopy reporting table

Supplementary Movie 1 - etMINFLUX recording of event sites as detected by a peak signal of Caveolin1-EGFP, with SM-STAR RED tracked in the MINFLUX channel.

Supplementary Movie 2 - etMINFLUX recording of event sites as detected by a peak signal of Caveolin1-EGFP, with DPPE-STAR RED tracked in the MINFLUX channel.

Supplementary Movie 3 - etMINFLUX recording of an event site as detected by an accumulation of Dynamin1 EGFP with STAR RED-membrane tracked in the MINFLUX channel, showing a budding endosome captured in the MINFLUX data.

Supplementary Movie 4 - etMINFLUX recording of an event site as detected by an accumulation of Dynamin1 EGFP with STAR RED-membrane tracked in the MINFLUX channel, showing a budding endosome captured in the MINFLUX data, present for 1 min.

Supplementary Movie 5 - etMINFLUX recording of an event site as detected by an accumulation of Dynamin1 EGFP with STAR RED-membrane tracked in the MINFLUX channel, showing a budding endosome captured in the MINFLUX data, present in an arrested state for a prolonged period of time.

Supplementary Movie 6 - etMINFLUX MultiROIFollow experiment mode example experiment, showing the following of multiple Gag accumulation event sites with MINFLUX tracking of STAR RED membrane and interleaved confocal imaging.

Supplementary Movie 7 - etMINFLUX recording of an event site as detected by an accumulation of Gag-EGFP with STAR RED-membrane tracked in the MINFLUX channel, showing a an initially flat membrane then continuously bulging at the event site.

Supplementary Movie 8 - etMINFLUX recording of an event site as detected by an accumulation of Gag-EGFP with STAR RED-membrane tracked in the MINFLUX channel, showing a budding viral particle then disappearing into a flat membrane as the Gag signal disappears.
